# Storage‐D: A user‐friendly platform that enables practical and personalized DNA data storage

**DOI:** 10.1002/imt2.168

**Published:** 2024-01-21

**Authors:** Xiaoluo Huang, Junting Cui, Wei Qiang, Jianwen Ye, Yu Wang, Xinying Xie, Yuanzhen Li, Junbiao Dai

**Affiliations:** ^1^ Shenzhen Key Laboratory of Synthetic Genomics Guangdong Provincial Key Laboratory of Synthetic Genomics, Key Laboratory of Quantitative Synthetic Biology, Shenzhen Institute of Synthetic Biology, Shenzhen Institutes of Advanced Technology, Chinese Academy of Sciences Shenzhen China; ^2^ School of Biology and Biological Engineering South China University of Technology Guangzhou China; ^3^ Shenzhen Branch Guangdong Laboratory of Lingnan Modern Agriculture, Genome Analysis Laboratory of the Ministry of Agriculture and Rural Affairs, Agricultural Genomics Institute at Shenzhen, Chinese Academy of Agricultural Sciences Shenzhen China

**Keywords:** codec platform, DNA data storage, Storage‐D, “Wukong” algorithm

## Abstract

Deoxyribonucleic acid (DNA) has been suggested as a very promising medium for data storage in recent years. Although numerous studies have advocated for DNA data storage, its practical application remains obscure and there is a lack of a user‐oriented platform. Here, we developed a DNA data storage platform, named Storage‐D, which allows users to convert their data into DNA sequences of any length and vice versa by selecting algorithms, error‐correction, random‐access, and codec pin strategies in terms of their own choice. It incorporates a newly designed “Wukong” algorithm, which provides over 20 trillion codec pins for data privacy use. This algorithm can also control GC content to the selected standard, as well as adjust the homopolymer run length to a defined level, while maintaining a high coding potential of ~1.98 bis/nt, allowing it to outperform previous algorithms. By connecting to a commercial DNA synthesis and sequencing platform with “Storage‐D,” we successfully stored “Diagnosis and treatment protocol for COVID‐19 patients” into 200 nt oligo pools in vitro, and 500 bp genes in vivo which replicated in both normal and extreme bacteria. Together, this platform allows for practical and personalized DNA data storage, potentially with a wide range of applications.

## Highlights

- Deoxyribonucleic acid (DNA) data storage exhibits remarkable advantages, such as high density and long lifespan, and is suggested to be one of the most promising media for coping with future data storage crises.
- A user‐friendly platform, “Storage‐D” was developed, which enables users to store any format of practical data with personalized choice.
- Specifically, a new algorithm, named “Wukong” was developed, which contains a sizeable collection of codec pins that enables encoding data into any DNA sequence with considerable privacy.
- The “Wukong” algorithm shows overall better performance than earlier algorithms in encoding a suitable length of DNA sequence matching downstream biochemical working flow for in vitro and in vivo storage.
- The tool provides an open‐frame for integrating other codec algorithms and can be easily connected to commercial DNA synthesis and sequencing platform for building a complete pipeline for practical data storage into DNA. The web server and codes of the platform are available at http://storage.dailab.xyz:16666/ and https://github.com/DNAstorage-iSynBio/Storage-D/, respectively.

## Graphical abstract

Deoxyribonucleic acid (DNA) has been suggested as a very promising medium for data storage in recent years. Although numerous studies have advocated for DNA data storage, its practical application remains obscure and there is a lack of a user‐oriented platform. Here, we developed a DNA data storage platform, named Storage‐D, which modularized essential functions for DNA data storage and provided personalized codec choices for users. A new codec algorithm called “Wukong” was specially designed and integrated into the tool, which outperforms previous algorithms in key practical application considerations. By connecting to commercial DNA synthesis and sequencing platform with “Storage‐D,” “Diagnosis and treatment protocol for COVID‐19 patients” was successfully stored in DNA both in vitro and in vivo. This platform allows for practical and personalized DNA data storage, potentially with a wide range of applications. The web server and codes of the platform are available at http://storage.dailab.xyz:16666/ and https://github.com/DNAstorage-iSynBio/Storage-D/, respectively.

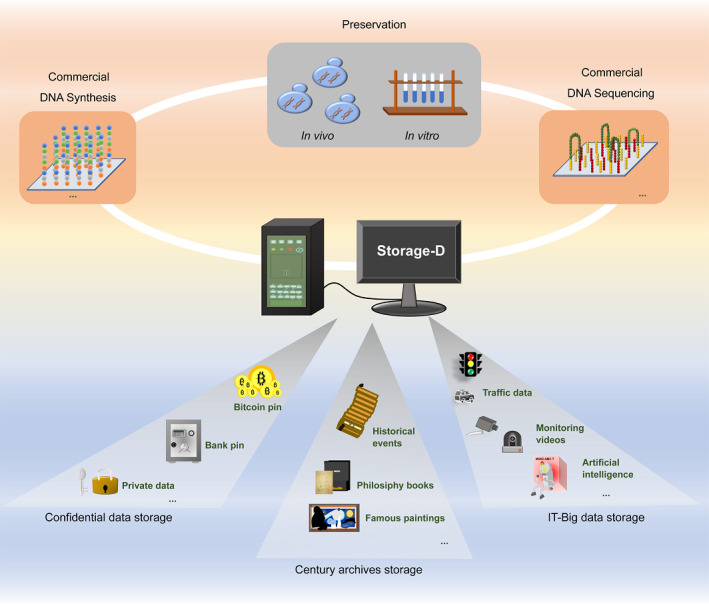

## INTRODUCTION

With the development of modern society, global data are accumulating explosively, and it is estimated to reach 163 ZB by 2025 [1]. Traditional data centers built from magnetic tape or silicon‐based storage medium may not satisfy the future data storage demand due to huge space or energy consumption, high cost of maintenance, and so forth [2, 3, 4]. Therefore, developing a new medium for data storage is urgent.

Among different polymers, deoxyribonucleic acid (DNA), as the natural carrier of genetic information, encodes the blueprint of life. In recent years, DNA has shown its potential as a data storage medium [2, 3, 4]. It is of ultrahigh density, with 6–7 orders of magnitude higher than the conventional magnetic tape or silicon‐based storage medium [2, 3, 4]. It also shows remarkable stability. Data can be archived in DNA for thousands of years under proper conditions [5]. Moreover, data stored in DNA can be copied with high fidelity by biochemical replication at a relatively low cost [6, 7, 8]. DNA also works as a superior candidate for encrypted data storage and can be hidden both in vitro and in vivo [2, 3, 4, 9, 10].

Although DNA data storage has been proposed since the 1960s, it did not receive much attention until the work published by Church et al. [11] and Goldman et al. [12], in which chip‐based DNA synthesis and next‐generation sequencing technology were employed in DNA data storage. Later, Grass et al. [5] showed that DNA encapsulated in silica can potentially preserve data in Zurich (9.4°C) for 2000 years and at the Global Seed Vault (−18°C) for more than 2 million years. Subsequently, Erlich and Zielinski demonstrated a physical density of DNA data storage of 215 PB/g DNA, which was orders of magnitude higher than the previous work [13]. These studies provided earlier qualitative data on how long and how much information DNA can retain. Furthermore, whereas previous work saved relatively tiny amounts of data in DNA, Microsoft's work explored the storage of over 200 MB data into manufactured oligo‐pool, pushing the possibilities of DNA for large‐scaled data storage usage [14]. Koch et al. also embbed DNA into functional materials that can be used to make any shape of objects, stored 1.4 MB video in DNA embedded in plexiglass spectacle lenses, and sequenced data from a tiny piece of plexiglass [15], which connected DNA data storage with daily life. In addition, a recent work by Chen et al. stored digital files in 254 KB synthetic chromosome that were stably replicated in yeast cells for 100 generations, suggesting a possible application of in vivo DNA data storage for massive data distribution [16]. While more and more work demonstrated the excellent properties of DNA as a data storage material, DNA data storage starts to reach the point of practical application.

The major DNA data storage process involves data codec, DNA synthesis, and DNA sequencing. Data codec achieves the conversion of binary digital information to A/T/C/G sequence. DNA synthesis writes the A/T/C/G sequence information into the synthesized DNA. DNA sequencing reads the sequence information from the synthesized DNA [2, 3, 4]. As the bridge between computer information and DNA sequence, data codec plays the most critical role in DNA data storage. A simple codec method works by mapping [00, 01, 10, 11] to [A, T, C, G], with a coding potential of Shannon limitation of 2 bits per nucleotide (bits/nt). However, owing to numerous repeats of 00, 01, 10, or 11 in the binary information extracted from figures, texts, audios, or videos, this codec method creates many homopolymer runs and high or low GC regions in the encoded sequence, which might strongly affect downstream DNA synthesis, amplification, or sequencing [17, 18], thereby leading to DNA data storage failure. Therefore, it is necessary to establish a robust codec method, which can satisfy the constraints in the biochemical experiments, while maintaining a high coding potential and data recovery fidelity.

Earlier algorithms proposed by Church et al. [11] and Goldman et al. [12] controlled the homopolymer runs in the encoded DNA sequences, but did not well control the GC content of the encoded DNA sequences and showed a relatively low coding potential. The current most advanced algorithms, such as those proposed by Erlich and Zielinski [13] and Ping et al. [19], demonstrated reasonably high coding potential while controlling homopolymer runs and overall GC content in the encoded DNA sequence. However, these “proof‐of‐concept” studies do not fully take into account practical application settings. One issue is that they do not consider regional sequence properties for encoded DNA sequences, which could be critical for data storage employing lengthy DNA fragments. Furthermore, these algorithms offer fixed coding manners and are challenging to fit for different end‐users, who may require a customized coding manner for data privacy consideration. These algorithms also lack personalized options, and their practical use is severely constrained by the absence of an effective web tool. It is suggested that such an online codec platform can translate any data into DNA sequence and vice versa, comparable to “Google Translator” to different languages. However, while the development of this online codec platform has been reported in public “News,” there is no comprehensive research yet [20]. Song and Zeng, for example, developed a very simple online window based on their algorithm, yet this tool has a very limited coding potential and can only turn text into a DNA sequence [7]. Furthermore, this online window is inadequately built and lacks conscious engineering integration of many specialized services being critical to DNA data storage, as well as extra optimization to provide user‐friendly practical data storage consideration. As a result, the goal of this study is to develop a codec platform that is accessible and friendly to end users and can be utilized for practical and personalized DNA data storage applications. We developed the “Storage‐D” web‐based codec platform, which modularized key codec functions and provided a customizable choice for practical data storage use. A novel algorithm called “Wukong” was developed and integrated into the platform, which employed a flexible encoding logic and was able to generate a large number of encoding rules that can be employed for various DNA data storage demands. Our platform should largely benefit DNA data storage research and application in various scenarios.

## RESULTS

### Interface and personalized selection of Storage‐D

**Figure 1:**
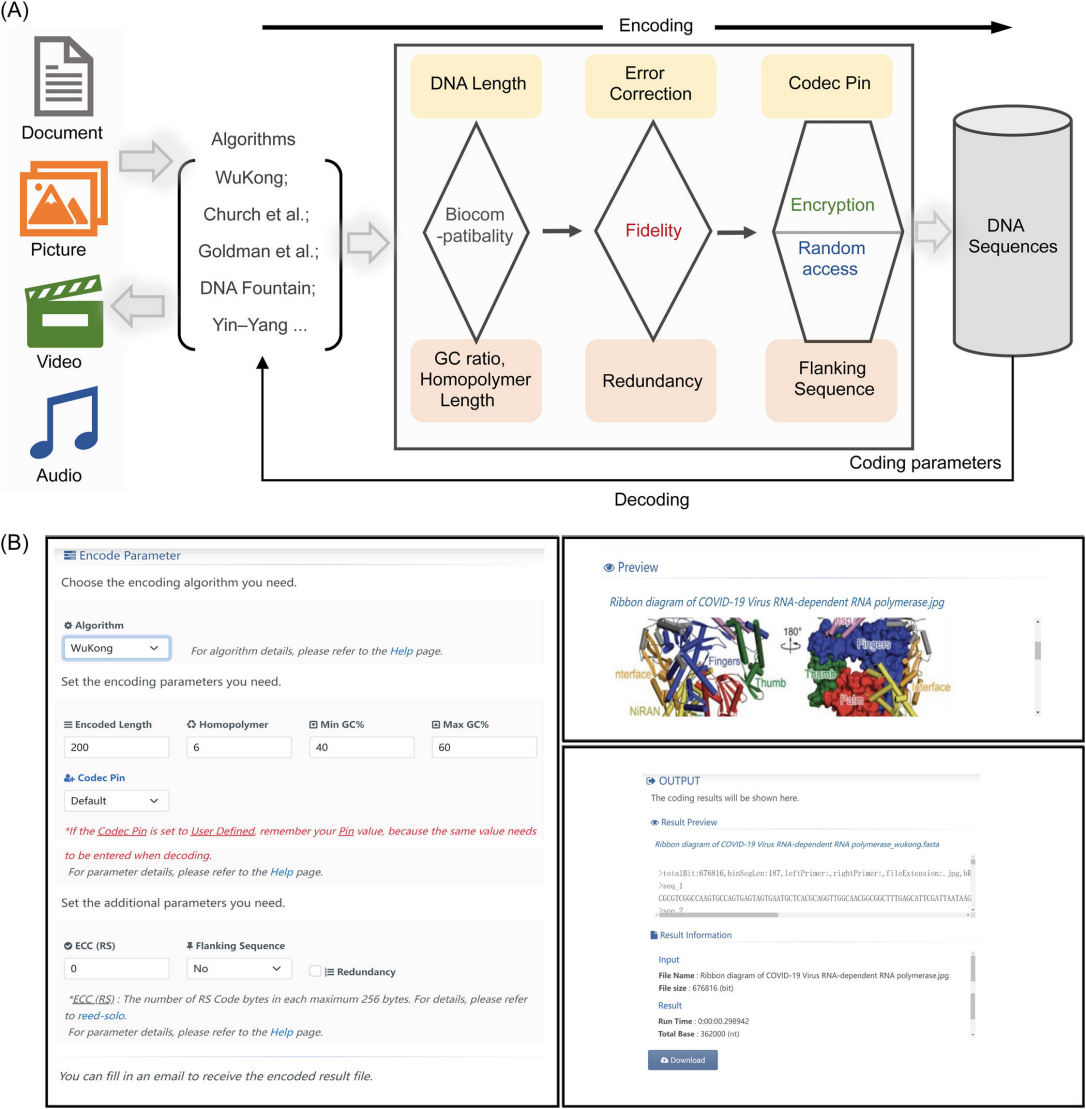
The working flow and interface of Storage‐D. (A) The codec working flow of Storage‐D. Storage‐D software supports four types of common text, image, audio, and video files as input, and provides “WuKong,” Church et al. [11], Goldman et al. [12], DNA Fountain [13], and Yin–Yang [19] coding algorithms for users to choose. As for the coding parameters, the user can customize the encoded DNA length, GC ratio, homopolymer length, error correction, redundancy, flanking sequence, and codec Pin. (B) The interface of Storage‐D. This indicates the conversion of an image of “RNA polymerase structure from COVID‐19 virus” [21] into DNA sequence.

The Storage‐D online tool contains data encoding and decoding functions, each with its page. The page is divided into input and output modules. The input module consists of “file upload” and “preview,” as well as “parameter selection,” and the output module includes a preview of the result and essential information related to the codec process (Figure 1).

For data coding, different images, text, audio, and video formats can be used as input and uploaded from the local files of the PC and Mobile devices. The uploaded data are visualized in a “preview” window, and the encoded DNA sequences in the FASTA format are shown in the output window. Internal parameters related to further data decoding are recorded on the first line. The output data can be also emailed to the user when the specific email box address is provided. In the decoding mode, the DNA sequence (e.g., obtained from DNA sequencing) in the same FASTA format as the encoding output can be converted to the desired image, text, audio, and video data and then downloaded. Notably, the uploaded data size is currently limited to 20 MB to avoid jamming the server. For larger files, users can download the Storage‐D program on GitHub and carry out the data codec locally.

Personalized codec features associated with “Wukong” algorithm including “Encoded length,” “Homopolymer,” “MinGC%,” “MaxGC%,” “Codec Pin,” “ECC (RS),” “Flanking sequence,” and “Redundancy,” is available for users to choose in Storage‐D. The “Encoded length” refers to the length of the DNA string used to encode the computational data. It directly relates to the DNA synthesis platform that users utilize to synthesize their encoded sequences. If users want to synthesize these sequences using a high‐throughput oligonucleotide synthesis platform, the default DNA length is set to 200 nt. “Homopolymer” refers to the maximum homopolymer length in the encoded DNA sequence, excluding the flanking sequence. “MinGC%” and “MaxGC%” refer to the average GC content in the encoded DNA sequence, excluding the flanking sequence. “ECC (RS)” refers to the error‐correction selection and “Redundancy” refers to whether one‐third of data redundancy is added to the encoded DNA sequence. To improve data fidelity, users can select these two parameters during their data encoding process. To randomly access the stored data, users can design their random‐access flanking sequence using Storage‐D. It is recommended that users should design their flanking sequence with a length of 20–25 nt. Notably, the pipeline implemented in Storage‐D is specifically used for designing random‐access flanking sequences towards the oligonucleotide pool or gene pool, which are the current most promising DNA media for large‐scale data storage. In addition, “Wukong” gives users the option of selecting personalized codec pins. Each codec pin contains two codec rules from the “Wukong” codec library. Storage‐D currently has 30,000 codec pins integrated for the user's selection. These are personalized pins for storing their DNA data, which will greatly increase the security of their data. Furthermore, the tool can email DNA sequence files to users or directly to a commercial company for DNA synthesis.

In addition, users can select previously established algorithms including those proposed by Church et al. [11], Goldman et al. [12], Erlich and Zielinski [13], and Ping et al. [19] to encode and decode their data. Notably, although some codec features are not present in their original publication as shown in Table 1, personalized designs, including “Encoded length,” “Homopolymer,” “MinGC%,” “MaxGC%,” “Codec Pin,” “ECC (RS),” “Flanking sequence,” and “Redundancy,” if possibly matched to the principle of the algorithm, is added into Storage‐D interface bound to these algorithms, providing a more flexible codec choice for these algorithms. With Storage‐D, users can easily choose any of the algorithms for data codec.

**Table 1:** Comparison between “Wukong” and other codec algorithms.^a^

General features	Details	Church et al.	Goldman et al.	Erlich et al.	Ping et al.	Wukong
Density	Coding potential^a^	1	1.58	1.98	1.95	1.98
Biochemical compatibility	Regional GC (%)	15−85	26−79	15−80	20−84	40–60 or defined
	Homopolymer	4	1	4	4	4 or defined
Data fidelity	Error correction	No	No	Yes	Yes	Yes or defined
	Redundancy	No	Yes	Yes	Yes	Yes or defined
Encryption	Codec pin	–	–	–	1536^b^	>20 trillion
Random‐access	Primer design	–	–	–	–	Yes or defined

*Note*: –, The feature design is not available in the algorithm.

^a^ The detailed features of algorithms proposed by Church et al. [11], Goldman et al. [12], Erlich and Zielinski [13], and Ping et al. [19] shown in the table maintain the features of their original publications. The coding potential of “Wukong” algorithm is calculated using the formula described by Ping et al. [19]. The rest of its coding potential is cited from Ping et al. [19].

^b^ The algorithm by Ping et al. [19] provided 1536 codec rules in their original publication, which might be used as codec pins in the future.

### The “Wukong” codec algorithm implemented in Storage‐D

**Figure 2:**
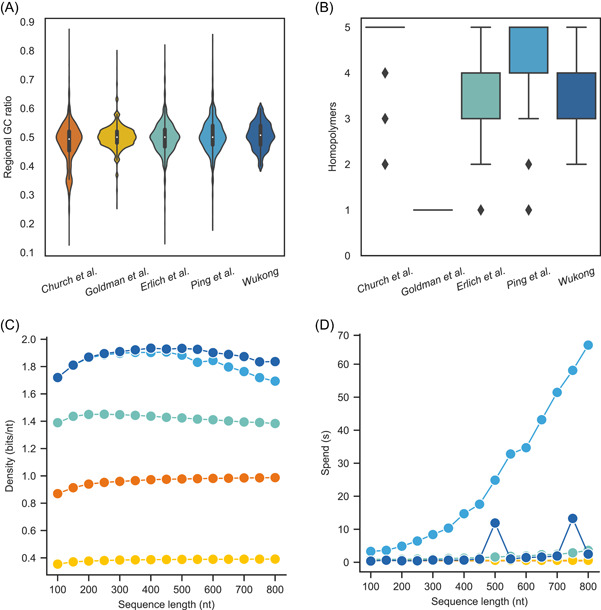
Encoding the DNA sequence with different lengths by algorithms implemented in Storage‐D. One image of 86,869 bytes was encoded into DNA sequence strings of 1000 nt by algorithms of Church et al. [11] (biochemical constraints: homopolymer ≤5 nt; indicated by orange‐red color), Goldman et al. [12] (indicated by gold color), Erlich and Zielinski [13] (biochemical constraints: homopolymer ≤5 nt, GC range of 0.4–0.6; indicated by turquoise color), Ping et al. [19] (biochemical constraints: homopolymer ≤5 nt, GC range of 0.4–0.6; indicated by sky‐blue color) and “Wukong” (biochemical constraints: homopolymer ≤5 nt, GC range of 0.4–0.6; indicated by deep‐blue color), while error correction, redundancy, and flanking sequence were not considered in the encoding. The 150 base pairs (bp) windows stepped by 140 bp from the 1000 bp encoded DNA sequence were used to analyze their GC content and homopolymer length. (A) The regional GC distribution of encoded DNA sequences by different codec algorithms. The number of 150 bp sequence windows falling into the given GC content was counted and plotted. The graph area within a particular part shows the frequency within that area. The greater the area is, the higher the frequency of the distribution represents. The white dots represent the median of the collection of data. (B) The regional homopolymer distribution of encoded DNA sequences by different codec algorithms. The number of 150 bp sequence windows falling into the given homopolymer length was counted and plotted. The black dot indicated outliers. (C) Practical encoding density by converting data into DNA sequence with 100–800 nt by “Wukong” and other algorithms. The density is defined as the amount of coded data divided by the total number of bases. (D) The running time of each algorithm encodes the data into DNA sequence with different lengths.

Coding potential is an important attribute for codec algorithms in DNA data storage since it directly relates to the amount of data that a specific amount of DNA can retain and, ultimately, the cost of DNA data storage. In comparison to other published binary codec algorithms, the “Wukong” algorithm currently has the highest coding potential of 1.98 bits/nt, which is the same as the “DNA Fountain” algorithm proposed by Erlich and Zielinski [13] and slightly higher than the “Yin–Yang” algorithm proposed by Ping et al. [19] (Table 1). Furthermore, although having the same coding potential, “Wukong” outperforms “DNA Fountain” in data recovery (Figure S1). “Wukong” also shows much higher practical coding density than “Yin–Yang” codec algorithm while encoding different lengths of DNA and it encodes equivalent lengths of DNA at a noticeably faster rate than the “Yin–Yang” codec approach (Figure 2C–D).

Long single‐nucleotide homopolymer runs typically result in mistakes during DNA synthesis and sequencing [22, 23, 24], which are thought to impact the decoding of data stored in DNA. Previous methods often limited it to a predetermined level. This may not be appropriate in some cases, such as nanopore sequencing, which prefers homopolymer runs of less than 4 nt. As a result of its flexible coding, “Wukong” is able to adjust the homopolymer run length below different nucleotides, such as 3, 4, or 5 nt (Figure S2). This should allow it to be used in a variety of scenarios.

The regional GC content has a considerable influence on polymerase chain reaction (PCR) amplification during the random‐access and DNA sequencing steps of DNA data storage [25]. Regional high or low GC content is generally not acceptable for long DNA fragment synthesis, while regional high or low GC and long homopolymer run have a particular impact on gene assembly from oligonucleotides [26]. The previous algorithms including “DNA Fountain” and “Yin–Yang” algorithms considered controlling the GC concentration only at the whole DNA level. This could result in significant GC bias at the regional sequence level (Table 1, Figure 2A). Notably, “Wukong” introduces a special design for checking the GC content and homopolymer runs within 150 nt windows, which enables “Wukong” to design long gene without regional biochemical constraints (Figure 2B). In particular, in accordance with the “Wukong” algorithm's flexible logic in the conversion of binary information to DNA sequence, it encoded DNA sequence falling into any ideal GC standard ranging from a relatively wide GC range of 35%–65% to a reasonably strict GC range of 45%–55% (Figure S2). This permits “Wukong” to be matched to different DNA synthesis and sequencing methods, while their sequence limits differ. Furthermore, “Wukong” can encode DNA with a GC content, which corresponds to genome GC of biological organisms (Table S1). This may allow “Wukong” to be suitable for in vivo DNA data storage.

It's noteworthy that “Wukong” offers flexible logic for data codec, allowing it to quickly produce over 20 trillion codec rules. This gives users a wide range of coding options. While prior work struggled to generate appropriate coding methods for DNA data storage, this codec library provides an unprecedented number of coding methods, which should largely free up much effort in data codec. While each coding process can utilize a pair of codec rules for data codec, this is especially crucial for data encryption. For example, different users can encode their data using different codec pins. Alternatively, different codec pins can also be used as markers to distinguish different types of data stored in DNA. Given the huge codec library of “Wukong” algorithm, it should be widely used in a variety of tailored DNA data storage scenarios.

Taken together, “Wukong” outperforms other codec algorithms in terms of overall performance, allowing it to be more robust in practical data storage than other algorithms.

### In silico evaluation of Storage‐D

To demonstrate the utility of Storage‐D, we first assessed its performance in encoding various types of data. The majority of computer data today consists of programs, texts, audio files, photos, and videos. Storage‐D can convert any format of these computer files into binary information in the coding process and vice versa in the decoding process. To test its running, different data with sizes ranging from about 3 KB to 7.7 MB were implemented into Storage‐D. As shown in Table S2, users can not only select “Wukong” algorithm, but also algorithms proposed by Church et al. [11], Goldman et al. [12], Erlich and Zielinski [13], and Ping et al. [19] to encode their data, and correspondingly decode their data. Furthermore, Storage‐D can encode simulated data from 1 to 10^4^ KB with a relatively constant density and a relatively fast speed within 1 min (Figure S3) utilizing its developed “Wukong” algorithm. Data decoding can also be completed with great accuracy and in less than 1 min for data sizes from 1 to 10^4^ KB (Figure S3). Furthermore, the encoded DNA segments had considerably similar GC performance, with the majority of them convergent approximately 50% GC content (Figure S3). Overall, Storage‐D provides an efficient data codec platform for DNA data storage.

Given that current commercial DNA synthesis provides different lengths of DNA fragments (e.g., ~120 nt by column‐based oligonucleotide synthesis, ~300 nt by chip‐based oligonucleotide synthesis, and assembled genes ranging from ~200 base pairs [bp] to 10 KB), as a second example, we tested “Storage‐D” in encoding different lengths of DNA. As shown in Figure 2D, by Storage‐D, users can encode variable lengths of DNA based on their downstream DNA synthesis demand.

**Figure 3:**
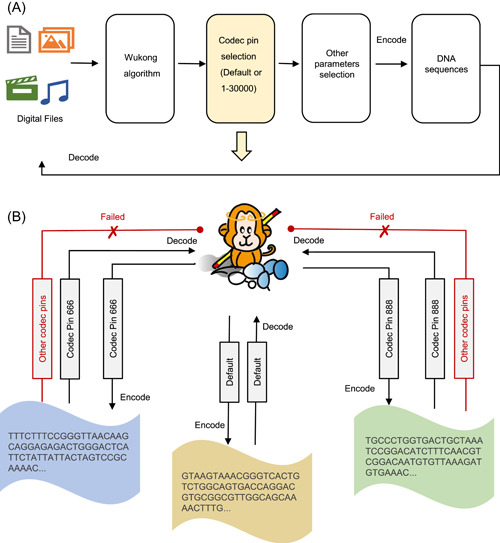
Encode data with different codec pins. (A) The working flow diagram of the encoding data into DNA with different codec pins. (B) An example to illustrate data codec based on the specialized pins. A cartoon figure showing that the main character “Wukong” from Chinese traditional myth is encoded into DNA sequences through the default choice or codec pin 666 and codec pin 888. For the same input file, if the user selects different codec pins, different encoding sequences are obtained. When decoding, if the wrong codec pin is entered, the correct result will not be obtained.

Furthermore, as a unique feature of Storage‐D, we tested its data encoding via various codec pins. Storage‐D currently integrated 30,000 codec pins, with significant differences, paired from the “Wukong” codec rule library. Users can either encode different data into different DNA sequences or the same data into different DNA sequences by selecting different codec pins (Figure 3A). Even though only the sum counts of each nucleotide at a given position are computed, different codec pins display noticeable encoding differences when data are converted into DNA sequence (Figure S4). This is consistent with the finding that the hamming distance between encoded sequences by various codec pins is significantly different (Figure S5). While different codec pins encode different DNA sequences, decoding the sequence by the wrong codec rule was impossible (Figure 3B). This provides an available approach for users to encrypt their data.

### Validation of “Storage‐D” by practical data storage

Given that DNA may potentially store data for millennia, we intended to store the precious experience of combating the COVID‐19 viruses during the last 3 years in DNA to pass this experience on to our descendants. The “Wukong” algorithm was then chosen to encode “*Diagnosis and treatment protocol for COVID‐19 patients Trial Version*” (seventh, eighth, and ninth versions with 333,360, 462,480, 416,280 bits, respectively) from the China National Health Commission and a classical traditional Chinese medicine book “*Treatise on Febrile and Miscellaneous Diseases*” (575,040 bits) into 1909, 2669, 2402, and 3319 DNA strings, respectively. Each string contains 200 bp, screened by biochemical constraints of “GC = 40%–60%” and “homopolymer length ≤4 nt.” Two bytes of the Reed‐Solomon (RS) error‐correction code were added to each binary string, and one‐third of the data redundancy generated by exclusive OR (XOR) conversion was added to the total binary data to improve data recovery fidelity. Every two binary strings segmented from the binary bits of the recorded data were encoded into a single DNA string, which was then screened. To access the data randomly, a well‐designed flanking sequence with 20 nt was added to each string at both ends (Figure S6A). The final encoded DNA sequences were synthesized as one oligonucleotide pool (Table S3).

**Figure 4:**
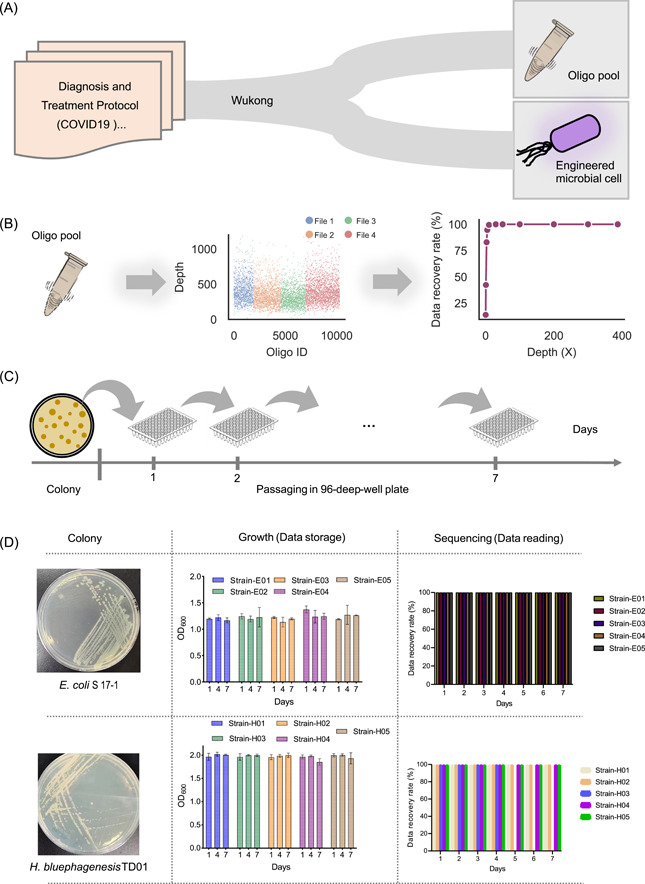
Validation of “Wukong” algorithms in vitro and in vivo. (A) Illustration of data storage in vitro and in vivo by “Wukong” algorithm. We synthesized the sequences encoded from the seventh, eighth, and ninth editions of the “Diagnosis and treatment protocol for COVID‐19” and the ancient Chinese medical work “Treatise” on Febrile and Miscellaneous Diseases by oligo pool, which was used for in vitro storage. In addition, we encoded and synthesized some traditional Chinese medicine formulas for treating COVID‐19 into genes stored in cells. (B) The oligo‐pool is read by sequencing. The sequencing depth of all the oligos is shown in the middle and the data recovery rate is presented on the right. (C) Illustration of data passage in vivo. (D) The data are stored in *Escherichia coli* S17‐1 and *Halomonas bluephagenesis* TD01 strain and stably replicated for 7 days. The growth state and data recovery rate obtained through sequencing are shown.

PCR amplification by random‐access primers (Table S4) towards each data file resulted in a clear band in agarose gel, suggesting that each data file could be successfully accessed (Figure S7A). Sequencing results showed that the sequencing depth of each file was very similar, indicating a high homogeneity for our library construction. The average sequencing depth of all the data files is 387× as shown in Table S5. Unfortunately, three oligos were completely missing from the sequencing data (Figure S7B), which might be attributed to a problem with DNA synthesis, DNA sequencing, or PCR amplification. Nevertheless, with the help of the RS code and redundancy added into each DNA string, a 100% data recovery was achieved while the sequencing depth was over 30×. Moreover, when the sequencing depth was cut to around 10×, over 99% of data recovery was achieved (Figure 4B). This result demonstrated the robustness of the “Wukong” codec algorithm for data recovery even at a low sequencing depth.

Because the preceding test is based on a synthesized oligo‐nucleotide pool of 200 bp that was kept in vitro, we also attempted to store data into assembled genes using “Storage‐D” and store them in vivo. As two example hosts for in vivo DNA data storage, we picked one typical bacterial host, *Escherichia coli*, and one extreme bacterial host, *Halomonas bluephagenesis*. Some of the data for in vitro storage above was encoded into 500 bp gene sequences (Table S6) by the “Wukong” algorithm (Figure S6B), following a biochemical constraint with a “homopolymer length ≤4 nt” and “GC content within the range on top of ±10% of the median GC of coding DNA sequences (CDSs) of the species” (Table S1). These gene sequences were synthesized and further transformed into each strain. As shown in Figure 4D, the bacterial strain containing the stored information showed stable growth, and the information was decrypted with 100% accuracy within 7 days of growth. The demonstration of data storage in *E. coli* and *H. bluephagenesis* by the “Wukong” algorithm implies that data can be stored in both normal and extreme bacteria.

Together, the achievement of storing “*Diagnosis and treatment protocol for COVID‐19 patients Trial Version*” and “*Treatise on Febrile and Miscellaneous Diseases*” into synthetic DNA and decoding them with high accuracy demonstrates the utility of “Storage‐D” for practical data storage.

## DISCUSSION

Storage‐D was mainly developed to provide users with a rapid, simplified, and feasible codec platform for studying DNA data storage. The efficient conversion of binary information into DNA sequence and vice versa may assist researchers to optimize the biochemical workflow associated with DNA data storage application, including random‐access PCR amplification, in vitro or in vivo preservation of DNA stored information as well as more efficient DNA synthesis and DNA sequencing pipeline. Storage‐D currently comprises a specifically devised “Wukong” algorithm and those algorithms proposed by Church et al. [11], Goldman et al. [12], Erlich and Zielinski [13], and Ping et al. [19]. In addition, Storage‐D is designed to provide users with variable parameter choices, including “encoded DNA length,” “GC” or “homopolymer runs,” “error correction,” “flanking sequence,” “redundancy,” and “codec pin.” This enables users to encode data based on their personalized interests. While the integration of other excellent algorithms is also worthwhile, Storage‐D defines a universal input and output style and provides an easy‐to‐use framework for integrating more robust algorithms in the future. Some algorithms, despite the fact that they might not be a universal algorithm or might be too similar to current algorithms, can be included in our platform to give consumers additional options [27, 28, 29, 30]. Furthermore, innovation algorithms that jumped over the binary conversion steps, which are currently in the proof‐of‐concept stage [31], could be implemented into the platform, if more and more tests on it are completed. More tailored considerations for various data file formats or certain types of data could also be included in the platform. Furthermore, data compression algorithms such as lossless compression and lossy compression [32] can be tested and implemented into the platform in the future. Overall, Storage‐D is a powerful tool for codec use in DNA data storage.

Storage‐D employs a specially designed algorithm, “Wukong.” The concept of “Wukong” emerged from our quest to devise a straightforward and intuitive method for encrypting individual data elements within the context of DNA data storage, utilizing distinct encryption keys, or “pins,” for each element. Although the computational program for data encryption has been developed over the years, none of them was designed to create a firewall during the conversion process between binary information and A/T/C/G sequences. This creates a gap concerning the data safety of DNA data storage. In this study, we created a pool of over 20 trillion codec rules of DNA data storage by mapping four binary bits to two nucleotides along with a permutation combination. Subsequently, we developed an algorithm that could select the codec “pin” during encoding. This approach can effectively fill in the safety hole regarding DNA data storage. Indeed, “Wukong” can be expanded further by mapping six binary bits to three nucleotides, eight binary bits to four nucleotides, and so on. This will create a larger codec rule library for data encryption if required for future research. Furthermore, 3, 4, 5… or more codec rules can be combined and integrated into the codec process following the pipeline of the “Wukong” algorithm. This approach enhances the feasibility of data encryption through codec rule combinations, thereby significantly mitigating the risk of inadvertent disclosure of sensitive information. Furthermore, without scarifying the coding potential, “Wukong” enables users to encode sequences with desirable GC and homopolymer length. Specifically, “Wukong” encodes DNA with even the GC ratio and short homopolymer in a 150 bp window, which should enable it to be suitable for more biochemical technologies including DNA synthesis, PCR amplification, and DNA sequencing used in DNA data storage.

**Figure 5:**
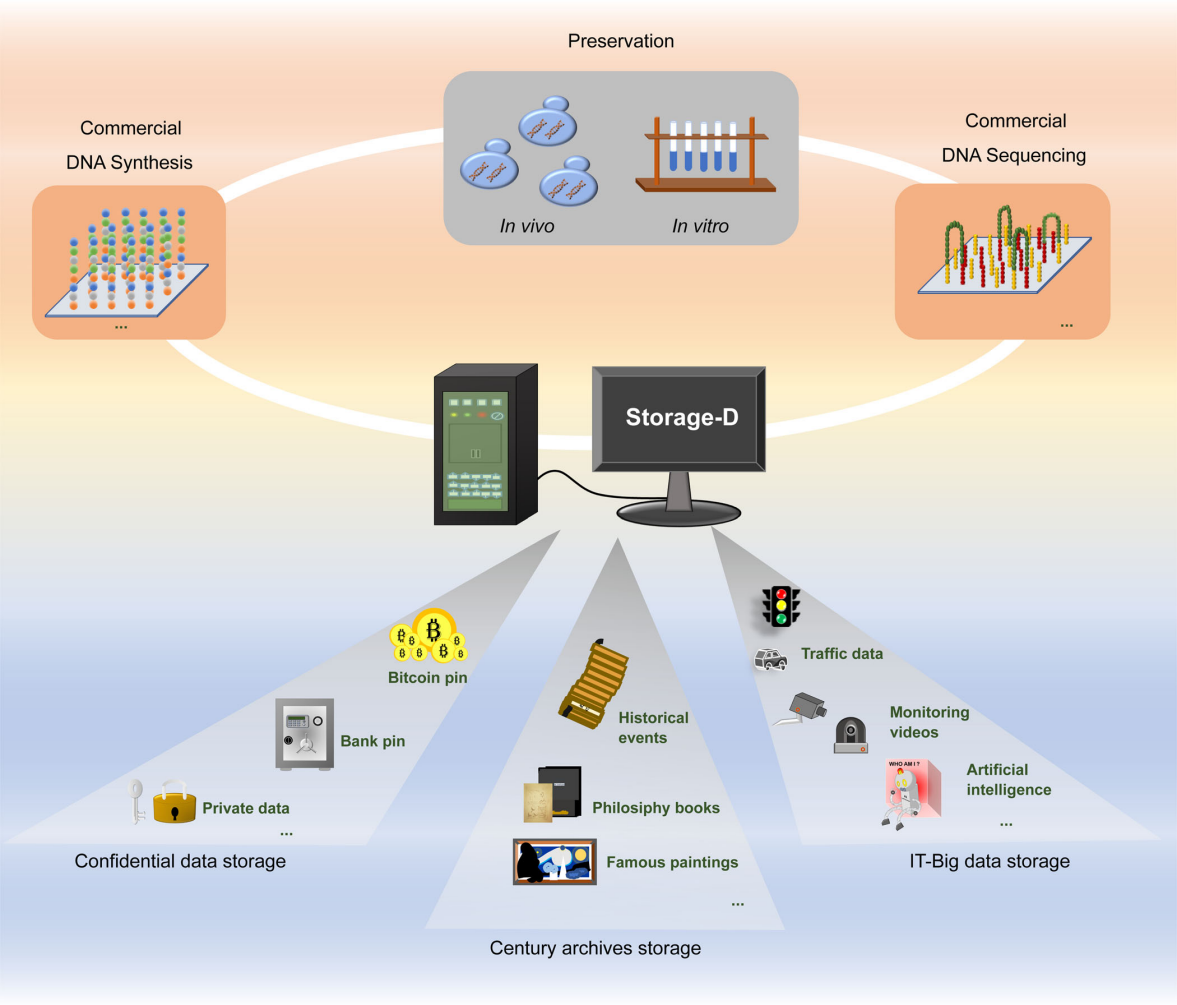
Storage‐D guided data storage: application envision. The integration of commercial DNA synthesis, in vivo and in vitro preservation, and commercial DNA sequencing with Storage‐D enables the development of a comprehensive DNA information storage application system. This advanced system can be utilized for secure data storage, long‐term archival purposes, and the efficient management of IT‐Big data.

Functioning as a codec platform that seamlessly integrates state‐of‐the‐art algorithms, its integration with commercial DNA synthesis and DNA sequencing platforms facilitates the streamlined establishment of a comprehensive DNA data storage pipeline. Therefore, storage‐D can be effectively utilized by researchers to store information into in vivo or in vitro DNA. On the basis of the advantages of Storage‐D, we postulate that it will be feasible for data storage application (Figure 5), for example, century archives storage. Under ideal circumstances, DNA has been proposed as a medium capable of preserving information for as long as 2 million years [10], a duration that surpasses that of any other storage medium known to date. The collective facets of human civilization, encompassing culture, historical events, and technological advancements, which necessitate transmission across generations, can be faithfully archived within DNA utilizing the Storage‐D platform (Table S7). Furthermore, this platform is equally suited for the secure storage of data from the past centuries, including personal interests, and serves as an excellent solution for highly confidential data storage. By using Storage‐D, data could be stored both in vitro as synthetic oligonucleotides, and in vivo into bacteria, plant, or animal cells. Moreover, the codec pins also build a confidential firewall between binary information and A/T/C/G sequences. As a result, confidential data such as bitcoin pins, bank card pins, and so on can be safely stored. Furthermore, Storage‐D has the potential to store big data in the future (particularly cold data) created by the internet, artificial intelligence, or cameras, given that the density of DNA is more than six orders of magnitude greater than that of today's most commonly utilized medium, such as hard disks and magnetic tape. Large data can be split into small scales, such as 20 MB, and processed using the Storage‐D platform for further storage into DNA. Future development can be also made to establish a framework for storing data on the PB scale, which might entail a thorough index design. Taken together, Storage‐D can play a significant role in future DNA data storage.

Nevertheless, the current cost of DNA data storage is relatively high, owing to the high DNA synthesis cost, which hinders its current application. The cost of storing 1 MB of data in a synthesized oligo‐pool exceeds $1500, based on prevailing commercial DNA synthesis rates. This cost structure has, to a certain extent, restricted the widespread adoption of Storage‐D, making it primarily viable for applications where price sensitivity is less critical. However, the DNA synthesis price has been decreasing owing to the development of super‐high‐throughput DNA synthesis technology and enzyme‐based DNA synthesis technology. To reduce the cost of DNA data storage, existing chip‐based DNA synthesis commercial suppliers, such as Twist Bioscience and CustomArray, are working to improve DNA synthesis throughput in a given size of chip. It is expected that if the throughput in one DNA synthesis chip increased from the current up to million oligos level to ~10^14^ oligos level (a palm‐sized chip [~10 cm × 10 cm] with ~10 nm scaled synthesis dot, similar to current electronic chip) with continuous optimization of the synthesis reaction, the DNA synthesis price would drop by more than 6–8 orders of magnitude, and the DNA data storage cost might start to approach the current hard‐disk cost. Similarly, if the price of enzyme‐based DNA synthesis falls in line with that of genome replication in one bacterial cell (overnight culture of 1 L Luria a‐Bertani [LB] media at a cost of ~$1 can yield ~10^9^ *E. coli* cells, each with an ~4 MB genome), the cost of DNA data storage might be even lower than that of a standard hard disk. Therefore, we speculate that Storage‐D should be used in broader data storage areas in the future.

## CONCLUSION

In summary, we developed a robust and user‐friendly codec platform, Storage‐D, for DNA data storage. With Storage‐D, users can encode and decode data with different formats with multiple well‐validated algorithms using personalized parameters. Particularly, it employs a newly developed codec algorithm, named “Wukong,” which contains a sizable collection of codec rules, allowing users to secure their stored data using a unique codec pin. On the basis of our findings, we believe that Storage‐D should largely benefit the DNA data storage use.

## METHODS

### The overall frame of Storage‐D

The development of Storage‐D was aimed at providing a user‐friendly platform for “translation” between computational data and A/T/C/G sequences. It modularized essential functions for DNA data storage including data codec, error correction, and random‐access, with multiple optional settings. To convert data into DNA sequences, a novel codec algorithm called “Wukong” was developed. It is capable of encoding DNA into diverse lengths with controlled homopolymer runs and regional even GC content while maintaining a high coding potential and offering a unique codec pin library. On top of it, an error‐correction function that was achieved by integrating “XOR” redundancy and error‐correction code was added. Finally, a specially devised “flanking sequences” generation process was used to achieve random‐access functionality. In addition, a sequencing analysis pipeline for decoding the DNA stored data was established and attached to this platform.

Apart from these, previously established algorithms, including those proposed by Church et al. [11], Goldman et al. [12], Erlich and Zielinski [13], and Ping et al. [19], were also integrated into the platform. The essential logic of these algorithms was published and the input and output styles were unified to adapt to the overall frame of the platform. Personalized designs, if possibly matched to the algorithm, were added to the Storage‐D online interface bound to these algorithms. Using Storage‐D, users can easily choose any of the algorithms for the data codec.

### The “Wukong” codec algorithm

The “Wukong” codec system starts from a codec rule library construction. A pool of 20, 922, 789, 888, 000 codec rules was generated from a permutation combination ($A_{16}^{16}$) of the codec mapping relationship between four binary bits and two nucleotides (Figure S8). Then, two coding rules were randomly selected from the library to encode the data information. Four binary bits were regarded as one coding unit, whether they came from a single binary data string or several distinct binary data strings. One coding rule was used in the (2n−1)th coding unit, and another was used in the 2nth coding unit. Notably, in this way, two binary fragments, three binary fragments, and four binary fragments could be encoded into one DNA sequence string, which then creates a rather free conversion between binary data and DNA sequence (Figure S9).

**Figure 6:**
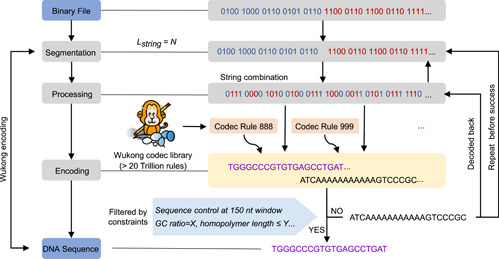
Principles of “Wukong” codec algorithm. The coding process of “Wukong.” Computational data are initially converted into binary information, and then segmented into the desirable length (*Lstring* = *N, “N”* represents any number). Two or more binary fragments are subjected to a random pairing process and are merged into one binary fragment, which is further transcoded into a DNA sequence with two selected codec rules. Subsequently, a DNA sequence with desirable properties (GC, homopolymer, …) is selected for further DNA synthesis. Sequences that do not meet the desirable standard are decoded back and put into the original binary fragment pool for a new round of encoding process.

To encode the data by “Wukong,” computer files were first converted into binary bits and then chopped into fragments with desirable lengths. Binary fragments were randomly selected and encoded using two rules selected from the “Wukong” codec reservoir. If the encoded DNA sequence met the preset GC and homopolymer runs, the selected binary fragments were deleted from the original binary fragment pool. The selected binary fragments that failed to generate desirable sequences were put back into the original pool (Figure 6). Occasionally, the selected binary fragments could not generate desirable sequences under 30 times repeats, and hence a virtual fragment was added to produce a desirable sequence. Eventually, DNA sequences with desirable sequence properties were designed.

### Error‐correction design

DNA synthesis, PCR amplification, and DNA sequencing create errors during DNA data storage [22, 23, 24]. Although the error rate of PCR amplification is low (generally below 1/100,000) [24], the error rate of commercial chemical oligonucleotide synthesis is as high as 1/2000–1/200 [22], and the DNA sequencing error can reach over 10% [23]. Although deep sequencing can generate folds of redundant reads for error correction, several errors still exist in the assembled DNA sequence for data decoding. Therefore, error correction is necessary if 100% data recovery is required.

One typical method for addressing errors in the stored data is the addition of an error‐correction code. While different error‐correction codes such as the Hamming code [33], RS code [5], and Low‐Density Parity‐Check code [16, 34] have been integrated into DNA data storage, the RS code is currently the most widely used. Here, we integrated the RS code for error‐correction design in Storage‐D. We provided users to add n (n≥1) bytes of the RS code while using “Wukong” algorithms. If users want to add RS code, provided bytes of RS code will be inserted after the index and redundancy (if user requested) have been added to each binary string encoded from the original data. We introduced a Python module “reedsolo” as validated by Erlich and Zielinski [13] and Ping et al. [19] before to compute and create RS code, as well as to carry out error correction and verification for decoding. These RS codes can detect up to *n* bytes of errors and correct up to *n*/2 bytes of errors. Notably, the error‐correction symbol used here is “1 byte,” which equates to “4 nucleotides” if encoded as DNA. This implies an *n*‐byte RS code can detect up to 4*n* base errors and fix up to 2*n* of them. For other integrated algorithms, except for the algorithm proposed by Goldman et al. [12], regardless of whether the RS code was integrated into their original publication, this RS code was also attached for their coding process. Hence, users can add n (n≥1) bytes of the RS code while using Storage‐D.

Occasionally, sequence loss or partial sequence loss could happen during DNA synthesis, PCR amplification, or DNA sequencing. Therefore, although the error‐correction code and redundant reads generated by DNA sequencing can revise several errors occurring during the biochemical experiments, complete data recovery might still be challenging. The introduction of information redundancy can solve this problem and enable DNA data storage with high fidelity. We introduced a redundancy design to the “Wukong” algorithm. Two contiguous fragments perform the XOR conversion to create a new fragment, generating one‐third of the redundancy in the total data (Figure S10). These could help users to decode their data with high fidelity, even when a considerable loss occurs during biochemical experiments. We also did the same for the algorithms described by Church et al. [11] and Ping et al. [19]. Nonetheless, given that Goldman et al. [12] introduced fourfold redundancy by encoding overlapping DNA sequences, and Erlich and Zielinski [13] employed the “DNA Fountain” code to generate a certain extent of redundancy in the encoding sequence, we did not add additional redundancy to these methods in Storage‐D due to their logic of creating redundancy throughout the encoding process.

### Random‐access design

The random‐access of data should also be considered during the codec process. Although several random‐access approaches were proposed [14, 35, 36, 37], the easiest way is to add flanking sequences at the pair end of the encoded DNA sequence, which can be further amplified by PCR. In 2018, Organick et al. [14] stored 35 different files with over 200 MB data in synthesized oligonucleotide pools and successfully recovered a separate file using a PCR‐based random‐access approach. To access the data randomly, flanking sequences that can be amplified by PCR were designed by the following procedure. First, the well‐developed oligonucleotide designing tool Primer3 program [38, 39] was employed to create a pool of flanking sequences using generated random sequences as the template. We limited the designed flanking sequences to have a GC content of 40%–60% and a melting temperature value of 58–70°C. To avoid possible unspecific amplification by random‐access primers towards encoded DNA sequence, the pool of flanking sequences was screened. Those flanking sequences with six nucleotides at the 3′ end that are identical to any 5′ end of the encoded DNA sequence or reverse complemented coded DNA sequence were removed. To further avoid cross pairing between random‐access primers and different flanking sequences, the remaining flanking sequences undergo a homologous analysis towards the encoded DNA sequence library by basic local alignment search tool (BLAST) [40], from which a pair of flanking sequences with the lowest score were selected and added at the pair end of the encoded sequence (Figure S11). The program of this procedure can be combined with any algorithm implemented in Storage‐D. Notably, given that the speed of the Primer3 program for designing the primer pool is relatively slow, a predesigned pool with ~20,000 sequences was implemented into Storage‐D. These sequences are also cross‐checked by homologous analysis and Hamming‐distance calculation towards each other, ensuring their suitability for downstream working flow.

### Encoded DNA length design

The current DNA synthesis technology normally produces DNA fragments below 10 KB. For commercial DNA synthesis, DNA of different lengths is generally offered at different prices. Therefore, to provide users with a choice of designing DNA fragments of different lengths, Storage‐D employs a specially designed pipeline to achieve this. The DNA length was defined as the sum length of the data payload region, index region, error‐correction region, and random‐access adaptors for “Wukong” and algorithms proposed by Church et al. [11] and Ping et al. [19]. For the algorithm proposed by Erlich and Zielinski [13], the DNA length was defined as the sum length of the data payload region, seed region, error‐correction code region, and random‐access flanking sequences. For the algorithm proposed by Goldman et al. [12], the DNA length was designed in terms of the internal logic of the algorithm (see Supporting Information text for more details). While the length of the index and error‐correction code varies, the final encoded DNA length equals or approaches the length that the user types into Storage‐D.

### Validation of Storage‐D platform by practical data storage

The Storage‐D platform was validated both in vitro and in vivo. For in vitro storage, four data files with 1,787,160 bits were encoded into 10,299 oligonucleotide sequences by the “Wukong” algorithm. The encoded DNA sequence was synthesized on the high‐throughput oligonucleotide synthesis platform (Twist Bioscience). A lyophilized pool of oligonucleotides (124 ng) was mailed from San Francisco, USA to Shenzhen, China at ambient temperature. To amplify the synthesized oligo‐pool, random‐access primers (Table S4) corresponding to the flanking sequences of each DNA string were synthesized at Tsingke using a column‐based oligo‐synthesis platform and purified using an oligonucleotide purification cartridge.

To mimic the real DNA data storage use, the synthesized oligonucleotide pool was first diluted to 10^8^ molecules/µL. To retrieve information, each file was amplified from the oligonucleotide pool by designated primers corresponding to a random‐access flanking sequence of each file. The PCR mixture and procedure were set up while a clear band was seen from agarose gel electrophoresis. About 2 µL of diluted oligonucleotide pool was amplified using a Q5 High‐Fidelity DNA Polymerase (NEB) in a total reaction volume of 50 µL with the following conditions: 98°C, 25 s; 65°C, 5 s with 25 cycles. Then, PCR products of each file were mixed by a ratio corresponding to its oligonucleotide numbers and sequenced on the Illumina MiSeq Platform (see Supporting Information text for detail).

For the analysis of the sequencing data of the amplified PCR products, paired‐end reads were first assembled by PEAR v0.9.11 [41]. Unassembled and low‐quality reads were discarded. Then, assembled sequences were chosen and filtered based on random‐access flanking sequences. Next, the quantity and base quality of the output sequencing reads from the last step were recorded. Reads with over 80% bases with quality values lower than Q30 were eliminated. The lengths of all the reads were then examined, and the reads that shared the same length and with the highest counts were preserved. Afterward, the sequences were first sorted by quality and then by quantity. Each sequence was granted a score based on its order number. BLAST [40] analysis was performed to cluster all sequences, and the sequence with the lowest order score from each cluster was chosen as an output (Figure S12). Flanking sequence regions of the sequence pool were then removed, and DNA sequences were decoded based on the specific “Wukong” codec rule used for encoding the data. The RS code was then applied to correct the substitution error. After filtering and reordering binary fragments by the index region, the remaining errors were then corrected by one‐third of the redundancy introduced by the encoding process. Next, the binary information was converted to the original data file.

For in vivo storage, partial data files (from in vitro data storage) with 4560 bits were encoded into genes with 500 bp each and synthesized at Beijing Liuhe BGI. The synthesized genes were then transformed and reproduced into *E. coli* S17‐1 and *H. bluephagenesis* TD01 strains after being cloned into a broad‐host pSEVA framed vector (see Supporting Information text for detail) [42, 43]. To check for stability, the data‐stored strain was continuously transferred into a 96‐well plate. The *H. bluephagenesis* strain was cultured in an LB medium supplemented with 5 g/L sodium acetate anhydrous, 50 g/L sodium chloride, and 25 mg/L chloramphenicol, whereas the *E. coli* strain was grown in the LB medium containing 10 g/L glucose and 25 mg/L chloramphenicol. Genes were amplified from grown cells daily and PCR products were used for Sanger sequencing to decipher the gene sequence.

### Implementation of Storage‐D online tool

The Storage‐D platform contains both offline programs (https://github.com/DNAstorage-iSynBio/Storage-D/) and one integrated online tool (http://storage.dailab.xyz:16666/). The Storage‐D online tool was developed in Python, run on Ubuntu 18.04.4 LTS, and was adapted to both Personal Computer and Mobile ends. It was written in Django, a Model‐View‐Controller‐based web frame (https://www.djangoproject.com/). The front end of Storage‐D employs a Bootstrap framework (http://getbootstrap.com/) for designing the overall style. It involves a management module and a display module. The management module contains file uploading, encoding, decoding, and results downloading parameters. The display module contains an uploaded file display and a downloaded file display. The back of Storage‐D contains a codec module and a file management module. Moreover, Storage‐D keeps an open function for the integration of additional strategies in the future.

## AUTHOR CONTRIBUTIONS

Xiaoluo Huang and Junbiao Dai designed the “Wukong” codec method. Junting Cui, Wei Qiang, and Yu Wang wrote and optimized the codes for the tool. Wei Qiang carried out the in silico experiments for the study. Jianwen Ye and Xinying Xie carried out the in vivo validation experiments. Yuanzhen Li did the PCR experiments and sequencing library construction. Xiaoluo Huang, Wei Qiang, Junting Cui, and Jianwen Ye prepared figures and tables for the manuscript. Xiaoluo Huang, Wei Qiang, and Junbiao Dai wrote the manuscript. Junbiao Dai and Xiaoluo Huang supervised the study. All authors performed the data analysis, read, and approved the final manuscript.

## CONFLICT OF INTEREST STATEMENT

Xiaoluo Huang and Junbiao Dai have applied for a patent about “Wukong” algorithm with application No. CN202011343923.9. Other authors have no competing interest.

## Supporting information


**Figure S1:** The relationship between effective reads ratio and file recovery rate.
**Figure S2:** Encoding sequence with different GC and homopolymer length by “Wukong” codec algorithm.
**Figure S3:** Different sizes of computational generated data with size of 1 to 10^4^ KB were used as original data to test the performance of Storage‐D.
**Figure S4:** Encoding the data with different codec pin.
**Figure S5:** Heatmap of Hamming distance between encoded DNA sequences by different codec Pins.
**Figure S6:** The architecture of the encoded DNA sequence by “Wukong”.
**Figure S7:** Experimental validation of “Wukong” implemented in Storage‐D.
**Figure S8:** The mapping relationship between 0/1 bits and A/T/C/G sequence.
**Figure S9:** Illustration of encoding different binary strings into one DNA sequence by “Wukong”.
**Figure S10:** Schematic overview of redundancy generation.
**Figure S11:** The pipeline of random‐access flanking sequence design.
**Figure S12:** The pipeline of sequencing data analysis.

**Table S1:** Encoding data in terms of GC content of different species.
**Table S2:** Encoding different files into DNA sequence by Storage‐D.
**Table S3:** Oligo sequences.
**Table S4:** The random‐access primers used to amplify each file.
**Table S5:** The average sequencing depth for each file.
**Table S6:** Gene sequences.
**Table S7:** Encoding historical data into DNA sequences.

## Data Availability

Storage‐D is available at http://storage.dailab.xyz:16666/. The codes package for algorithms implemented in Storage‐D and the codes used for analyzing sequencing data from oligo‐pools are available at GitHub with the address: https://github.com/DNAstorage-iSynBio/Storage-D/. Supporting Information materials (methods, figures, tables, scripts, graphical abstract, slides, videos, Chinese translated version and update materials) may be found in the online DOI or iMeta Science http://www.imeta.science/.
