# Supplementary material for: Storage‐D: A user‐friendly platform that enables practical and personalized DNA data storage
Source: Imeta. 2024 Jan 21;3(2):e168. doi: 10.1002/imt2.168 (PMC11170965; doi:10.1002/imt2.168)
Supplement: Supplementary file 1 — Figure S1: The relationship between effective reads ratio and file recovery rate. Figure S2: Encoding sequence with different GC and homopolymer length by “Wukong” codec algorithm. Figure S3: Different sizes of computational generated data with size of 1 to 104 KB were used as original data to test the performance of Storage‐D. Figure S4: Encoding the data with different codec pin. Figure S5: Heatmap of Hamming distance between encoded DNA sequences by different codec Pins. Figure S6: The architecture of the encoded DNA sequence by “Wukong”. Figure S7: Experimental validation of “Wukong” implemented in Storage‐D. Figure S8: The mapping relationship between 0/1 bits and A/T/C/G sequence. Figure S9: Illustration of encoding different binary strings into one DNA sequence by “Wukong”. Figure S10: Schematic overview of redundancy generation. Figure S11: The pipeline of random‐access flanking sequence design. Figure S12: The pipeline of sequencing data analysis. [file IMT2-3-e168-s001.docx]

Supporting information to
“Storage-D: a user-friendly tool that enables practical and personalized data storage in DNA”

Xiaoluo Huang^1#*^, Junting Cui^1#^, Wei Qiang^1#^, Jianwen Ye^3#^, Yu Wang^1^, Xinying Xie^3^, Yuanzhen Li^1^, Junbiao Dai^1,2*^

^1^Shenzhen Key Laboratory of Synthetic Genomics, Guangdong Provincial Key Laboratory of Synthetic Genomics, Key Laboratory of Quantitative Synthetic Biology, Shenzhen Institute of Synthetic Biology, Shenzhen Institutes of Advanced Technology, Chinese Academy of Sciences, Shenzhen 518055, China

^2^Shenzhen Branch, Guangdong Laboratory of Lingnan Modern Agriculture, Genome Analysis Laboratory of the Ministry of Agriculture and Rural Affairs, Agricultural Genomics Institute at Shenzhen, Chinese Academy of Agricultural Sciences, Shenzhen, 518000, China

^3^School of Biology and Biological Engineering, South China University of Technology, Guangzhou, 510006, China;

# These authors contributed equally: Xiaoluo Huang, Junting Cui, Wei Qiang, Jianwen Ye

* Correspondence: [daijunbiao@caas.cn](mailto:daijunbiao@caas.cn) (Junbiao Dai) & [huangxl@siat.ac.cn](mailto:huangxl@siat.ac.cn) (Xiaoluo Huang)

**Contents:**

The logic for designing the encoded DNA sequence with different length

The Reed Solomon (RS) code applied for DNA data storage

Details of *in vitro* and *in vivo* experiments

Command line steps-encode and decode the data by “Wukong” algorithm implemented in Storage-D

Supplementary Figures S1 to S12 attached to the main text

## The logic for designing the encoded DNA sequence with different length

Storage-D designs the DNA sequence with desirable length in terms of the user’s request. The complete DNA fragment designed by Storage-D contains multiple functional regions following the specific algorithms’ logic.

For “Wukong” and algorithm proposed by “Ping *et al.* [1]”, if users enter a DNA sequence length of “L_DNA_”, then, L_DNA_ can be calculated as:

$$\begin{aligned} L_{DNA}=\left( l_{i}+l_{R}+l_{D} \right)\times S\times\left( 1/K \right)+2\times l_{A}\#\left( \text{1} \right) \end{aligned}$$

where $l_{i}$, $l_{R}$ and $l_{D}$ represent the binary length of index, the length of RS code and the length of data payload respectively, S refers to the number of binary fragments that one DNA sequence contained, $K$ is defined as the maximum binary bits that one nucleotide can represent, and $l_{A}$ represents the length of the random-access adaptors.

For “Wukong” algorithm, while “$S$ =2”, “$K$=2”, then, L_DNA_ can be simply determined as:

$$\begin{aligned} L_{DNA}-l_{R}=l_{i}+l_{D}+2\times l_{A}\#\left( \text{2} \right) \end{aligned}$$

Since $l_{A}$ is entered by users, to design the sequence with desirable length, the essential point is to assure the values of “$l_{i}$” and “$l_{D}$” _._ Given that “Wukong” algorithm introduces one bit for the recording of the “virtual fragments” added during the bio-constraints screening, the practical index length, $l_{i}^{'}$ for the data information is “$l_{i}^{'}$-1”. Further, “$l_{i}^{'}$”and “$l_{D}$” are calculated by the combination of following (3) and (4), from which the minimum value of $l_{i}^{'}$ and the maximum value of $l_{D}$ are obtained:

$$\begin{aligned} l_{i}^{'}+l_{D}=L_{DNA}-l_{R}-2\times l_{A}-1\#\left( 3 \right) \end{aligned}$$

$$\begin{aligned} 2^{l_{i}'}\times l_{D}\geq B_{total}\#\left( 4 \right) \end{aligned}$$

where $B_{total}$ represents the total binary bits of the input data files. Once “$l_{i}^{'}$” and “$l_{D}$ ” are assured, the input length of encoded DNA “L_DNA_” is achieved.

For “Yin-Yang” codec algorithms (4), the calculation is the same to “Wukong”. For algorithms proposed by “Church *et al. (*1)”, while “$S$=1”, “$K$=1”, “$l_{i}$” and “$l_{D}$” are determined by the combination of following Equation (5) and (6), from which the minimum integer value of l_i_ and the maximum integer value of $l_{D}$ are obtained:

$$\begin{aligned} l_{i}+l_{D}=L_{DNA}-l_{R}-2\times l_{A}\#\left( 5 \right) \end{aligned}$$

$$\begin{aligned} 2^{l_{i}}\times l_{D}\geq B_{total}\#\left( 6 \right) \end{aligned}$$

where $B_{total}$ represents the total binary bits of the input data files. Once “$l_{i}$”and “$l_{D}$” are assured, the input length of encoded DNA “$L_{DNA}$” is achieved.

For “DNA Fountain” algorithms (1), the logic is different from the three algorithms described above. When the users enter a DNA sequence length of “$L_{DNA}$”, then, $L_{DNA}$ could be calculated as:

$$\begin{aligned} L_{DNA}=\left( l_{BR}+l_{BD}+l_{BS} \right)\times8/2\#\left( 7 \right) \end{aligned}$$

where $l_{BR}$ refers to the byte-length of error correction code, $l_{BD}$ is defined as the byte-length of data payload and $l_{BS}$ represents the byte-length of the seed. In Storage-D, the seed length is fixed to be “4”, therefore, $l_{BD}$ is calculated by the following expressing, from which, the maximum integer value of $l_{BD}$ is obtained.

$$\begin{aligned} l_{BD}\leq\frac{1}{4}L_{DNA}-l_{BR}-4\#\left( 8 \right) \end{aligned}$$

Once $l_{BD}$ is assured, the input length of encoded DNA “$L_{DNA}$” is achieved.

For algorithm proposed by Goldman *et al*. (2), when the users enter a DNA sequence length of “$L_{DNA}$”, then $L_{DNA}$could be expressed as:

$$\begin{aligned} L_{DNA}\geq l_{Gi}+l_{GR}+l_{GD}+2\times l_{A}\#\left( 9 \right) \end{aligned}$$

where $l_{Gi}$refers to the index, $l_{GR}$ represents RS code added by Storage-D, $l_{GD}$ is defined as payload region, which should be multiple of 4, in terms of the original algorithm, and $l_{A}$ is the flanking sequences added by Storage-D. $l_{Gi}$ is calculated by the combination of Equation (10), (11), and (12), while the maximum value of $l_{GD}$ and $L_{DNA}$ are used.

$$\begin{aligned} l_{GD}\in\{4n,n\in N+\}\#\left( 10 \right) \end{aligned}$$

$$\begin{aligned} l_{GD}+l_{Gi}\leq L_{DNA}-l_{GR}-2\times l_{A}\#\left( 11 \right) \end{aligned}$$

$$\begin{aligned} 3^{l_{Gi}}\times l_{GD}\geq T_{total}\#\left( 12 \right) \end{aligned}$$

Where $T_{total}$ represents the total ternary bits of the data generated by Huffman transcoding; Once “$l_{Gi}$”and “$l_{GD}$” are assured, the input length of encoded DNA “$L_{DNA}$” is achieved.

## The Reed Solomon (RS) code applied for DNA data storage

The Reed Solomon (RS) code is a block-based error correcting code that has been widely used in data storage and digital communication. Briefly, RS code can be described as below. First, RS codes are designed based on the information length and the number of errors to be corrected, such as RS code (n, k, m), where the information length is “k”, the check length is “m”, and the code length is “n”. Then, the original information is divided into “a” blocks, each with a length of “k”, and corresponding check redundancies of length “m” are generated for these "a" information blocks. When errors are detected in the received information, they can be corrected using the check redundancies at the end of the code. In 2015, Grass *et al.* firstly introduced RS code to DNA data storage [2]. Specifically, in Grass et al.’s work, each two byte of digital file was mapped to three elements of the Galois Field of size47. Then, the original information was arranged into blocks of 594 x 30 elements. Redundancy and index were then added to generate a matrix block of 713 x 39 elements. RS codes were introduced to generate redundancy A of blocks of 119 x 30 elements, and redundancy B of blocks 713 x6 elements. In 2017, Erlich *et al.* added an RS code of 8 nt for every 128 nt of encoded DNA information in their proposed“DNA Fountain”encoded scheme [3]. A recent work by Ping *et al.* added 16 nt RS code to every 128 nt encoded DNA unit [1]. Given its history, RS code is currently widely accepted in the DNA data storage codec algorithms [4-6]. However, because RS code is based on finite fields, the length of encoded data blocks is restricted by finite field settings, thus the errors that a set of RS codes can correct are also limited. As a result, combining RS code with other redundant encoding methods can improve the error-correction ability of DNA data storage. Furthermore, there is no comprehensive comparison of RS code to other error-correction strategies, such as Hamming code, parity check code, BCH code, Reed Solomon (RS) code, Fountain code, Turbo code and LDPC code [7]. As a result, future development may concentrate on developing a much better error correction strategy for DNA data storage, which can be further integrated into the Storage-D platform to provide users with more options.

## Details of *in vitro* and *in vivo* experiments

### DNA amplification, sequencing library construction and sequencing

The synthesized oligo pool from Twist BioScience (USA) in a dry powder form was firstly diluted into 10^8 molecules/ul by ddH_2_O. Random-access primers as shown in Table S1 were used to amplify the four stored data file using Q5 High-Fidelity DNA Polymerase at the following conditions: 98˚C, 25 sec; 65˚C for 5 sec with 25 cycles. The resulting PCR products were purified using 1×Hieff NGS® DNA Selection Beads (Yeasen Biotechnology, China). The concentration of purified PCR products was measured by Qubit and then, they were mixed in a quantity (ng) ratio corresponding to the ratio of oligo numbers of different data files in the synthesized oligo-pool. Then, 100 ng DNA was taken and dissolved in 25 ul ddH_2_O for further library construction. The DNA sequencing library is constructed using NEBNext® Ultra™ II DNA Library Prep Kit (Illumina, USA) following its protocol. After steps of “end preparation”, “adaptor Ligation”, “cleanup of Adaptor-ligated DNA”, and “PCR Enrichment of Adaptor-ligated DNA”, the library is purified with (0.9×) Hieff NGS® DNA Selection Beads (Yeasen Biotechnology, China). To reduce the interference of possible primer dimers, we further purified DNA from last step by gel purification. Finally, 14.7 ng/ul DNA was used for further sequencing. After qualification check, the DNA sample is sequenced by Illumina-Miseq run on “Shenzhen Infrastructure for Synthetic Biology”.

### Plasmid construction and transformation for *in vivo* data storage

The plasmids were mainly constructed via Gibson Assembly. The DNA fragments that contain targeted storage information, and primers used for PCR amplification were synthesized by BGI (China). All of the nucleotide fragments for plasmid construction, including storage DNA fragments and vector backbone (pSEVA321), were generated through incorporation of PCR amplification by High-Fidelity DNA polymerase and gel extraction by commercial tool kits (Tiangen). Subsequently, single colonies harboring assembled plasmids were picked from the selection plates and verified by PCR and DNA sequencing for further test. Strain *E. coli S17-1* was used as host for plasmid construction and donor cell for plasmid conjugation to *Halomonas spp.*, respectively. Plasmids that contain targeted DNA storage information were tested in strains, including *E. coli S17-1* and *Halomonas bluephagenesis TD*, respectively.

## Command line steps-encode and decode the data by “Wukong” algorithm implemented in Storage-D

We offer the step-by-step pseudocodes in Python 3.10.4 for reproducibility:

Encoding

Python

>>> from StorageD.codec import WukongEncode

>>> encode_worker = WukongEncode(input_file_path="testFile/dna.jpg", output_dir="testResult/", sequence_length=200,max_homopolymer=4,min_gc=0.4, max_gc=0.6, rule_num=1, rs_num=0, add_redundancy=True,add_primer=True, primer_length=20)

>>> encode_worker.common_encode()

Decoding

Python

>>> from StorageD.codec import WukongDecode

>>> decode_worker=WukongDecode(input_file_path='testResult/dna_wukong.fasta',output_dir="testResult/", rule_num=1)

>>> decode_worker.common_decode()

## Supplementary Figures S1 to S12 attached to the main text


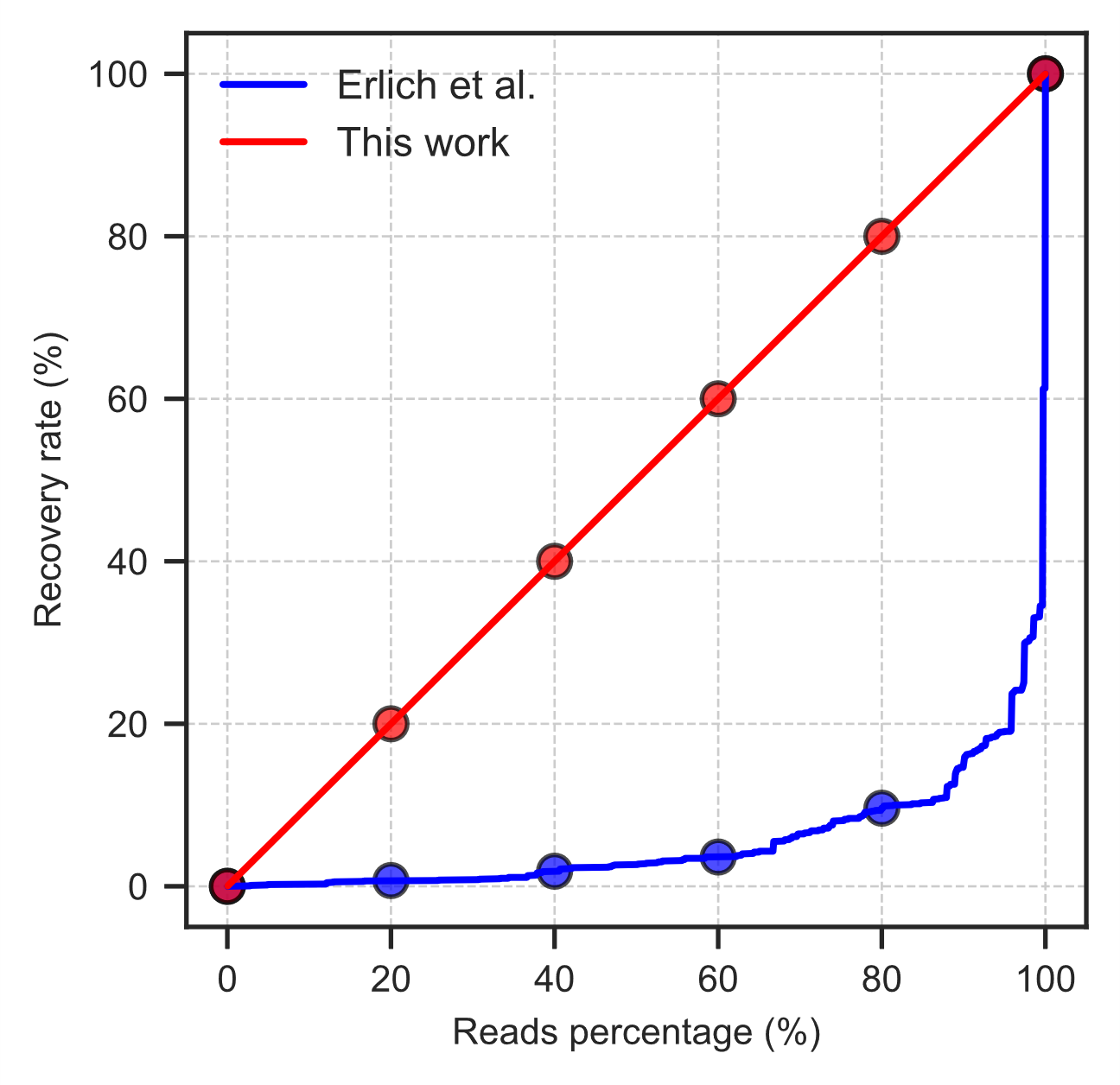


### Figure. S1 The relationship between effective reads ratio and file recovery rate.

Even if the reads ratio reaches 80%, the file recovery rate is still less than 20% by DNA Fountain. Nevertheless, the file recovery rate by “Wukong” increases in equal proportion to the effective reads ratio, which exhibits a better data recovery while the effective reads ratio is under 80%.


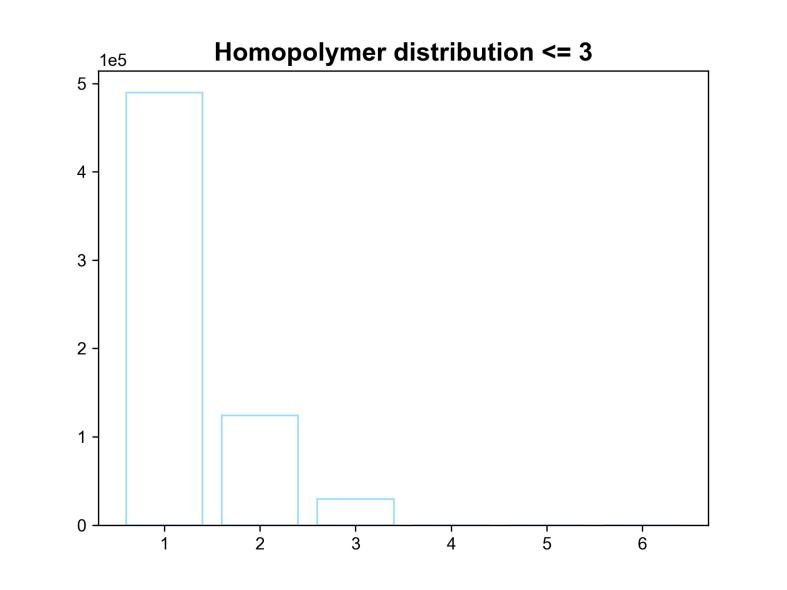

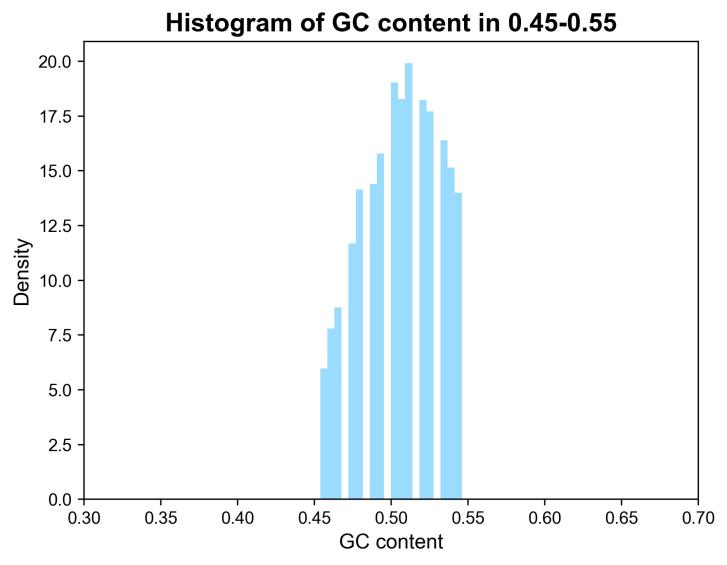

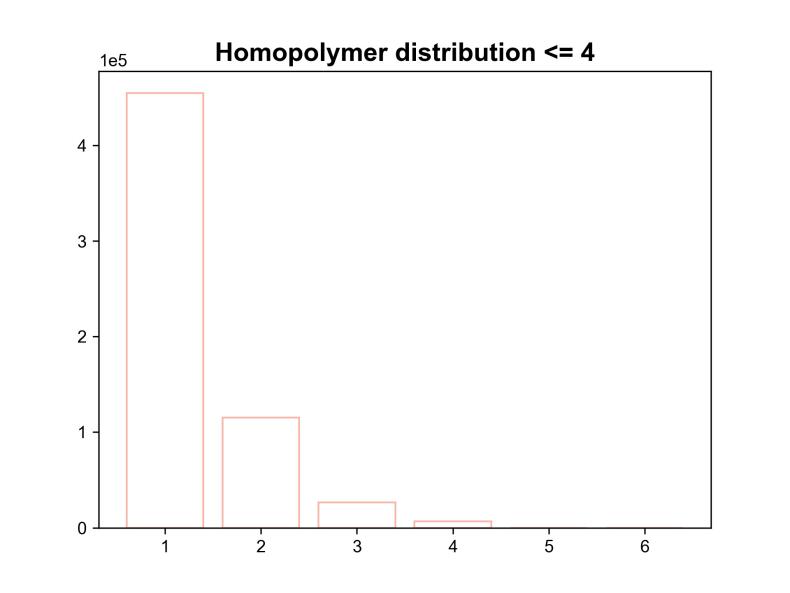

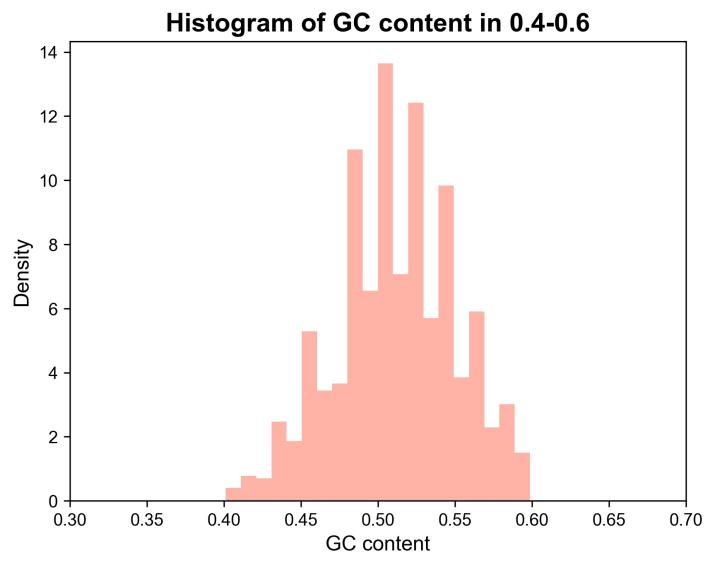

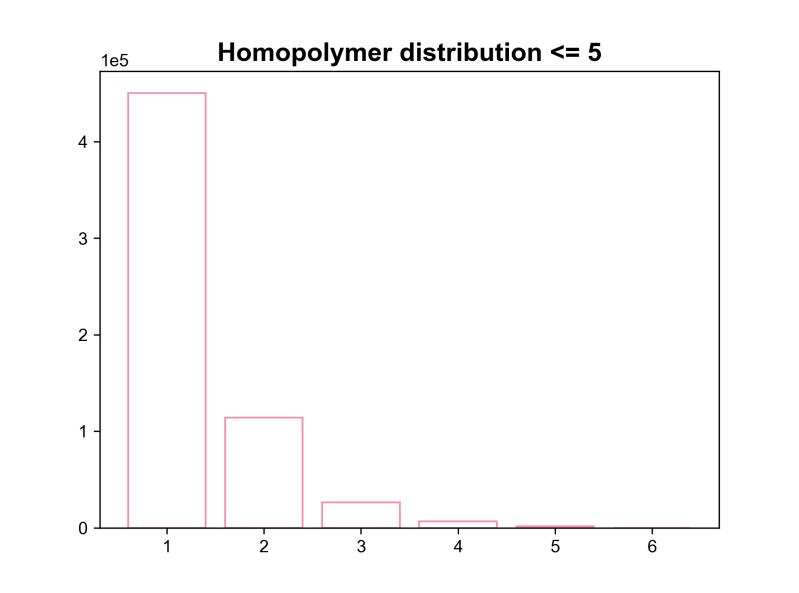

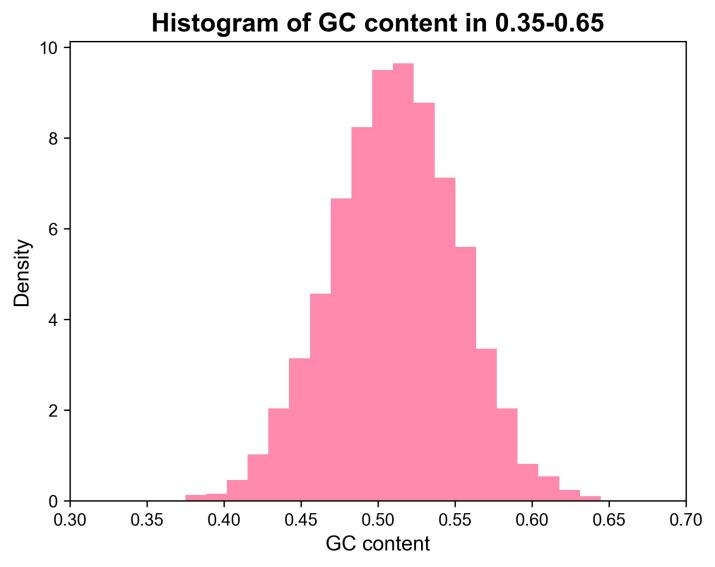


**A**

**B**

**C**

### **Figure S2. Encoding sequence with different GC and homopolymer length by “Wukong” codec** algorithm.

(**A**) Encoding the data into DNA sequence with GC content of 45% to 55% and single nucleotide homopolymer length ≤ 3 nt. (**B**) Encoding the data into DNA sequence with GC content 40% to 60% and single nucleotide homopolymer length ≤ 4 nt. (**C**) Encoding the data into DNA sequence with GC content of 35% to 65% and single nucleotide homopolymer length ≤ 5 nt. One image of 86,869 bytes is used as the data.


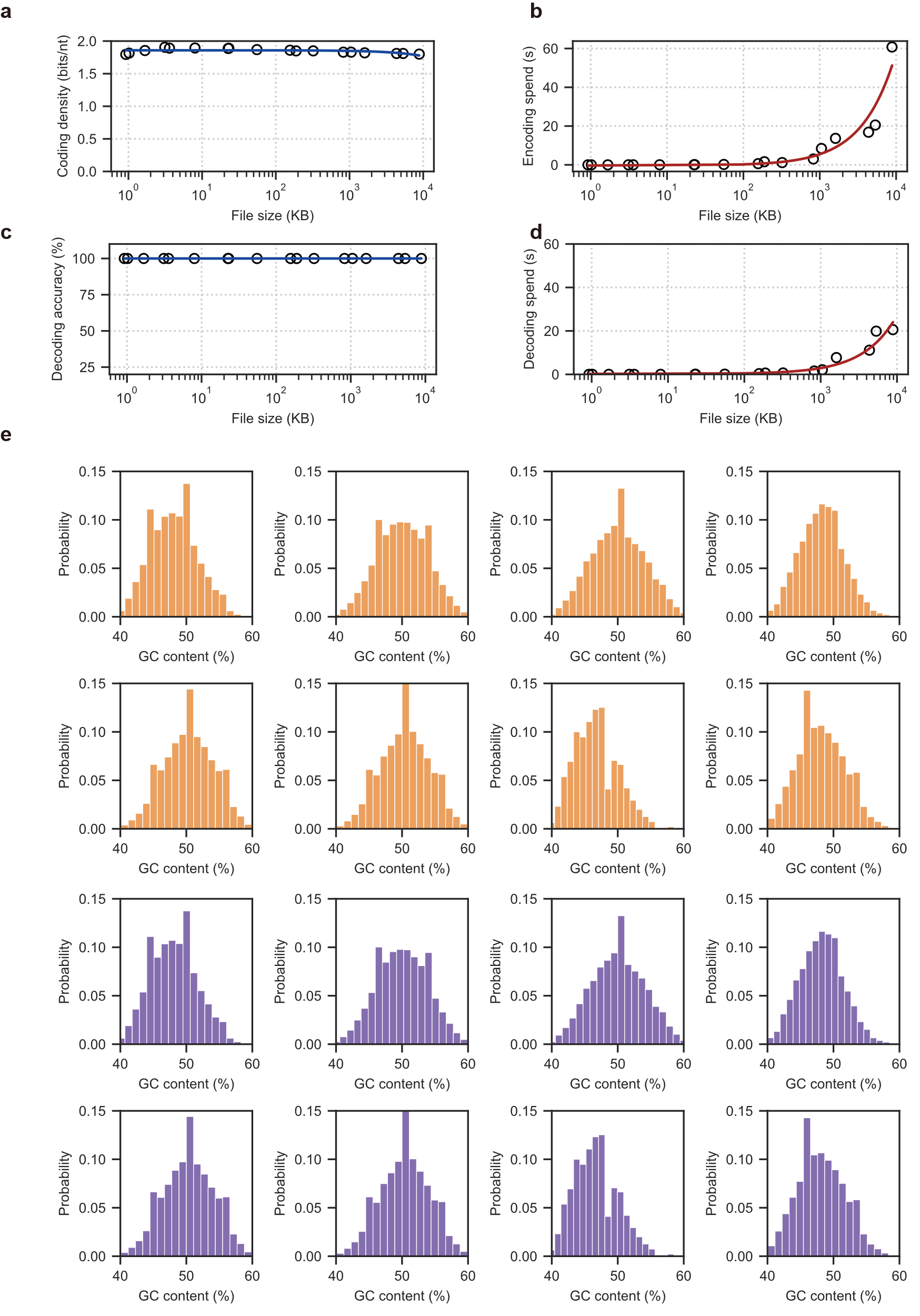


### Figure S3. Different sizes of computational generated data with size of 1 to 10^4^ Kb were used as original data to test the performance of Storage-D.

“Wukong” algorithm is selected to encode these data into DNA sequences with settings “GC of 0.4-0.6, homopolymers≤6, oligo length=200 nt”. a. The density performance of encoding data with different sizes. For different size of data, Storage-D encoded it with a nearly constant density. b. The time performance of encoding simulated data with different sizes. Three image files, “A Thousand Li of Rivers and Mountains partial.jpg”, “big fac.jpg”, and “Riverside Scene at Qingming Festival partial.jpg”, were proportionally scaled to produce 18 samples ranging in size from 1 Kb to 10^4^ Kb. The time increases as the data size increases while encoded in Storage-D platform. c. The decoding accuracy from encoded DNA sequence with different sizes by computer simulation. No error is introduced in the encoded DNA sequence. d. The time performance of decoding data from DNA sequences encoded from different size of data. e. The GC distribution of different data encoded by Storage-D. The orange color indicated the encoding results from texts (**Table S5**) of “A Dream in Red Mansions.txt”，“Journey to the West.txt”，“Mengxi Bitan.txt”，“Nine Chapters on Mathematical Procedures.txt”，“Romance of the Three Kingdoms.txt”，“Shih Chi.txt”，“the art of war.txt” and “The Water Margin.txt” and purple color indicated the encoding results from images (**Table S5**) of “A Thousand Li of Rivers and Mountains (partial).jpg”，“calligraphy of Liu Gongquan.jpg”，“calligraphy of Wang Xizhi.jpg”，“calligraphy of Yan Zhenqing.jpg”，“Riverside Scene at Qingming Festival (partial).jpg”，“Six Steeds of Zhao Mausoleum partial.jpg”，“The Night Revels of Han Xizai (partial).jpg” and “The statue of Fuxi and Nuwa.jpg”


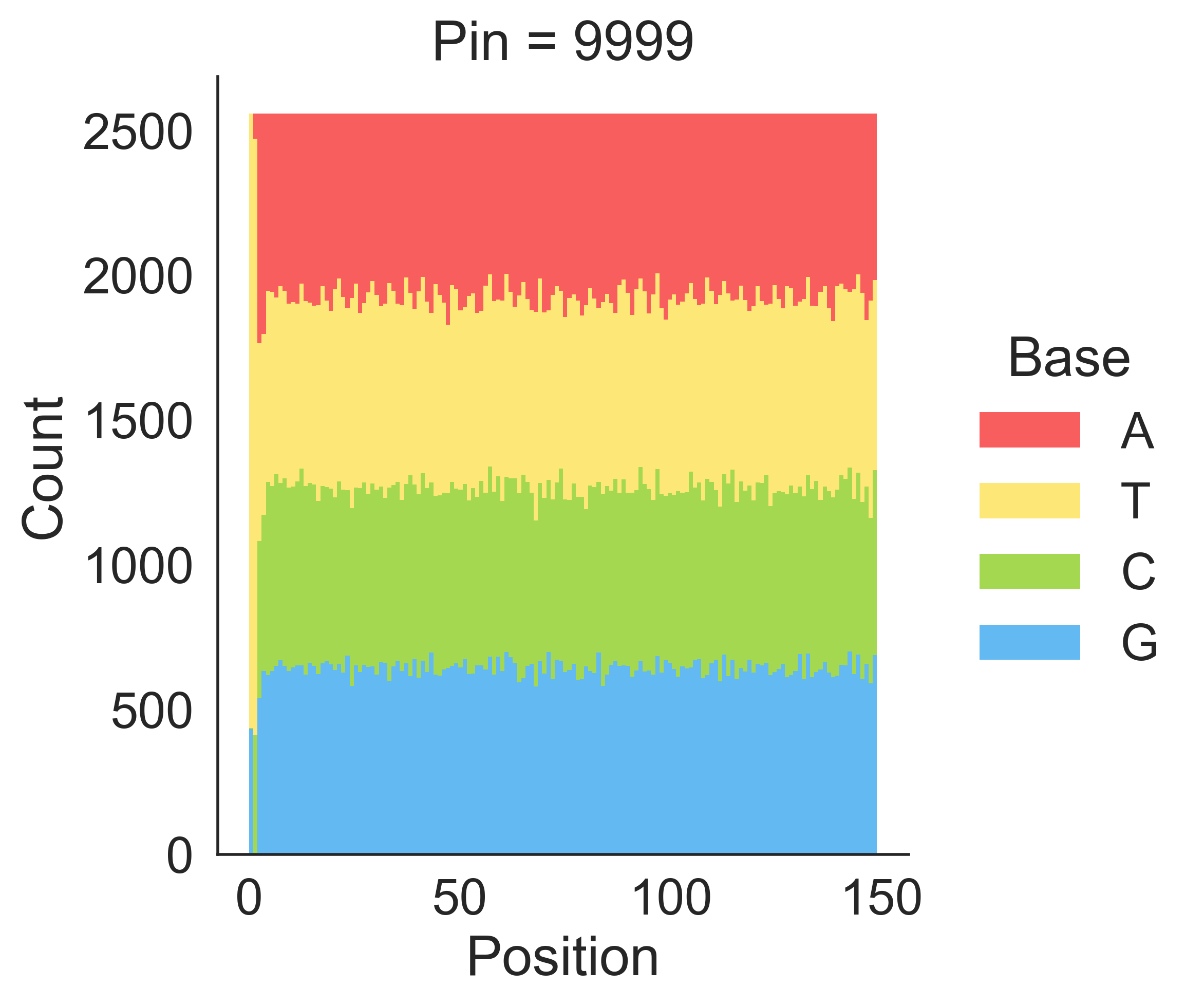

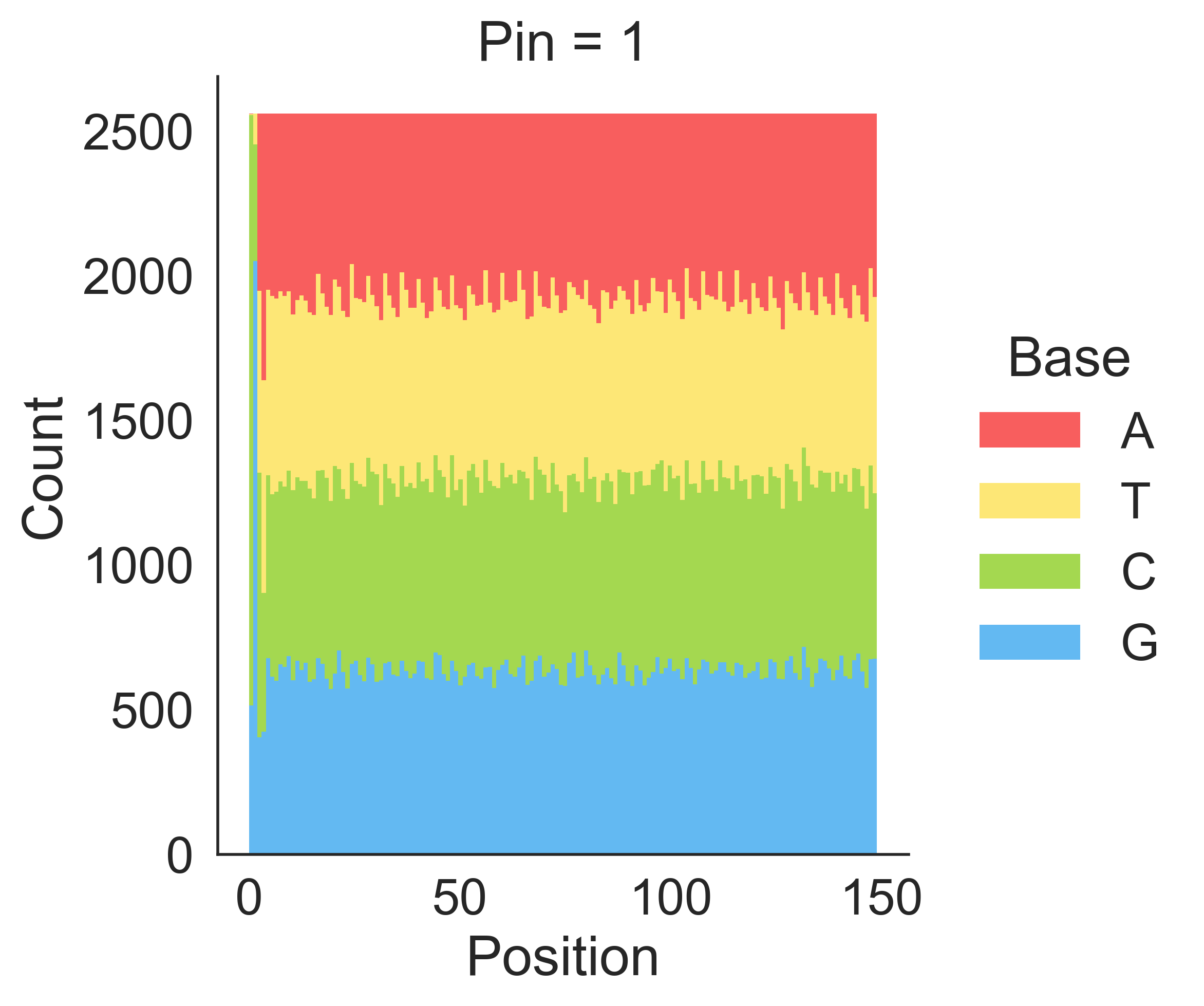

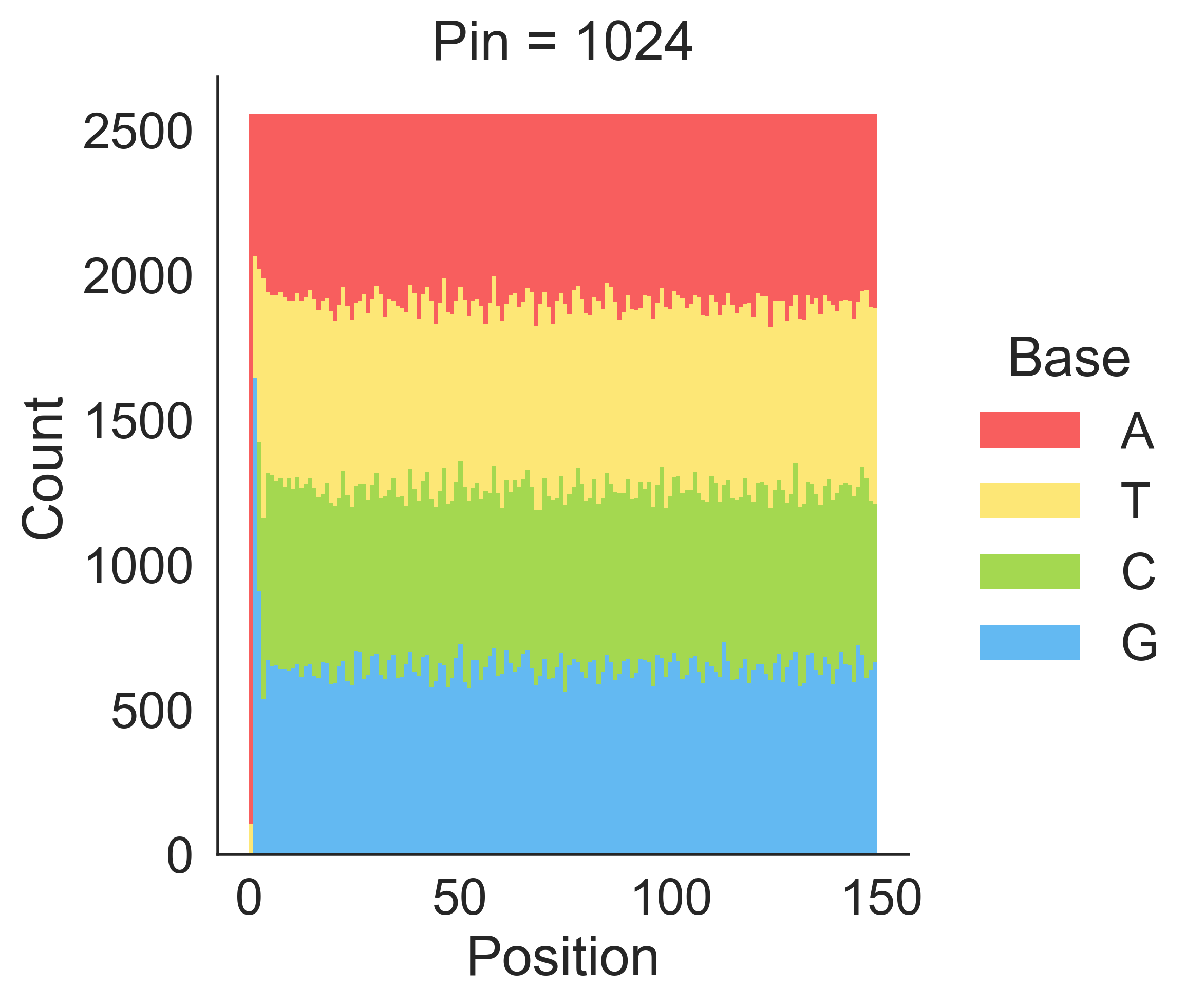

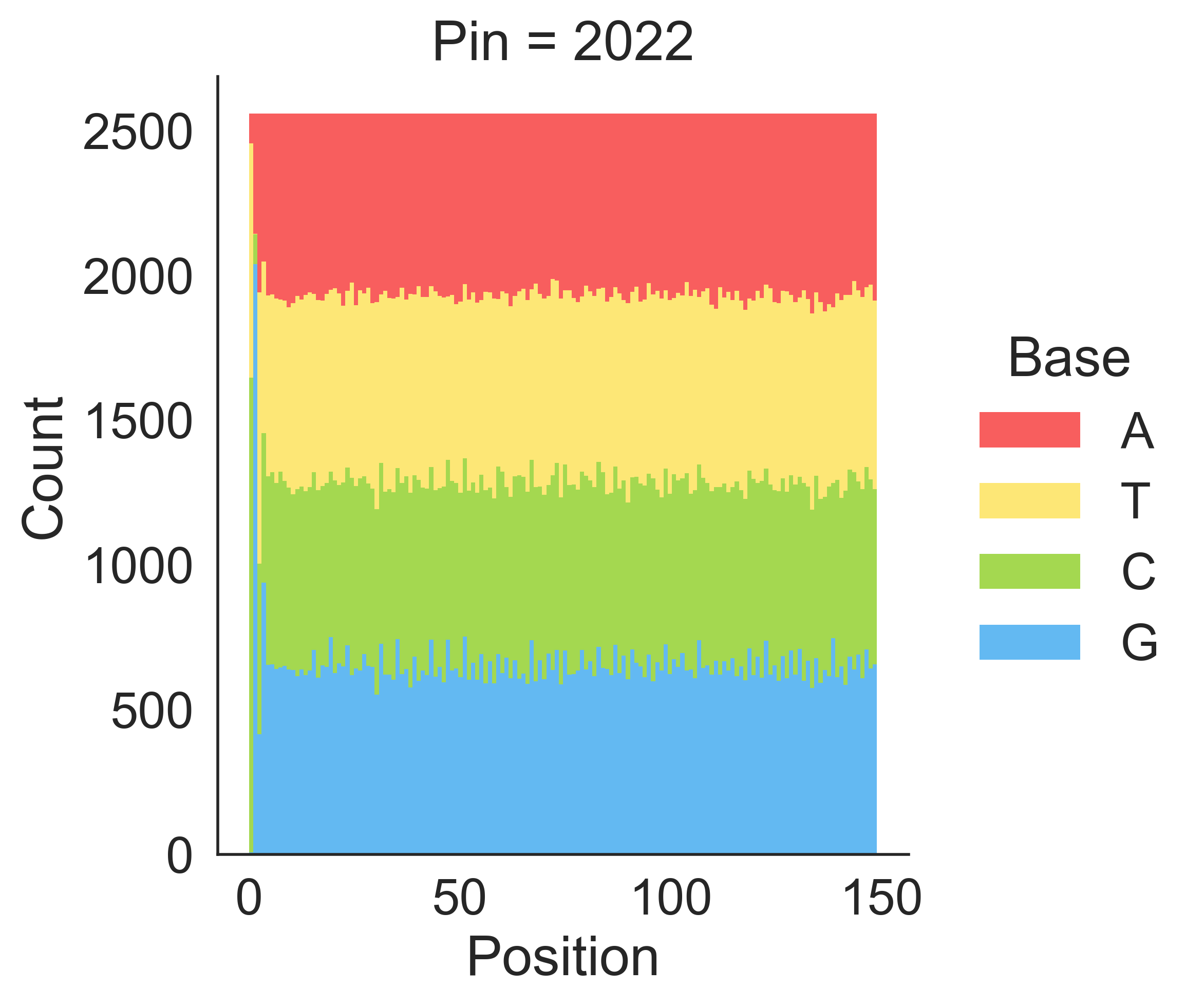


### Figure S4. Encoding the data with different codec pin.

The image of 86,869 bytes is encoded into DNA sequences with 150 nt by codec pin number of “1”, “2022”, “1024”, “9999”, by Storage-D using “Wukong” algorithm with biochemical constraints “homopolymer ≤ 4 nt, GC range of 40% to 60%”, while error correction, redundancy and flanking sequence are not considered in the encoding. The sum of each nucleotide at given position of the 150 nt sequence is counted.


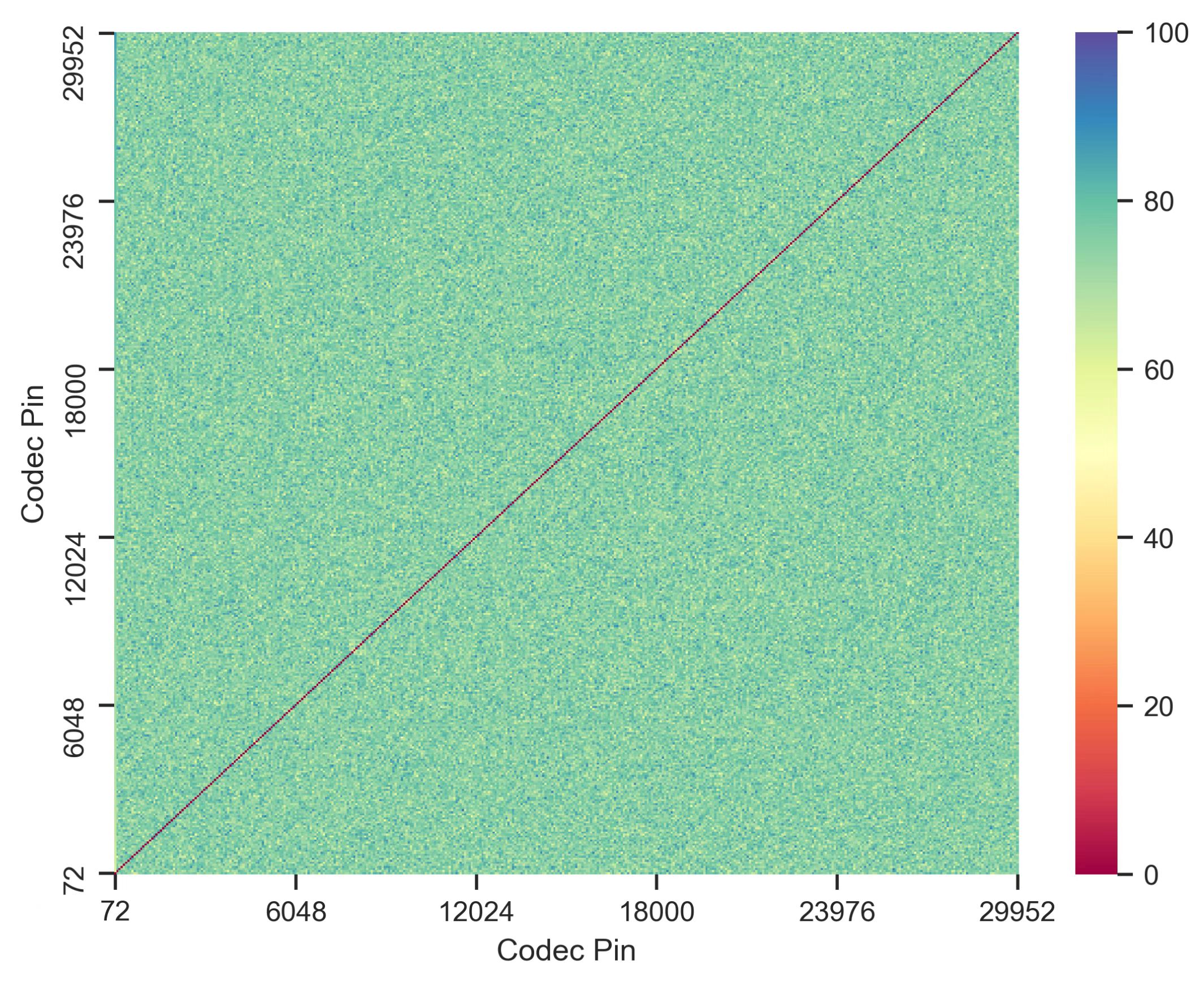


### Figure S5. Heatmap of Hamming distance between encoded DNA sequences by different codec Pins.

Color density indicates the difference of Hamming distance between the encoded DNA sequences by two codec pins, calculated with “100 x Hamming distance/the number of encoded nucleotides.” Codec pins with number “72n (n was integer and $\geq$1)” are employed to encode the image of 86,869 bytes into 200nt DNA sequences with biochemical constraints “homopolymer ≤ 6 nt, GC range of 30 % to 70%”.


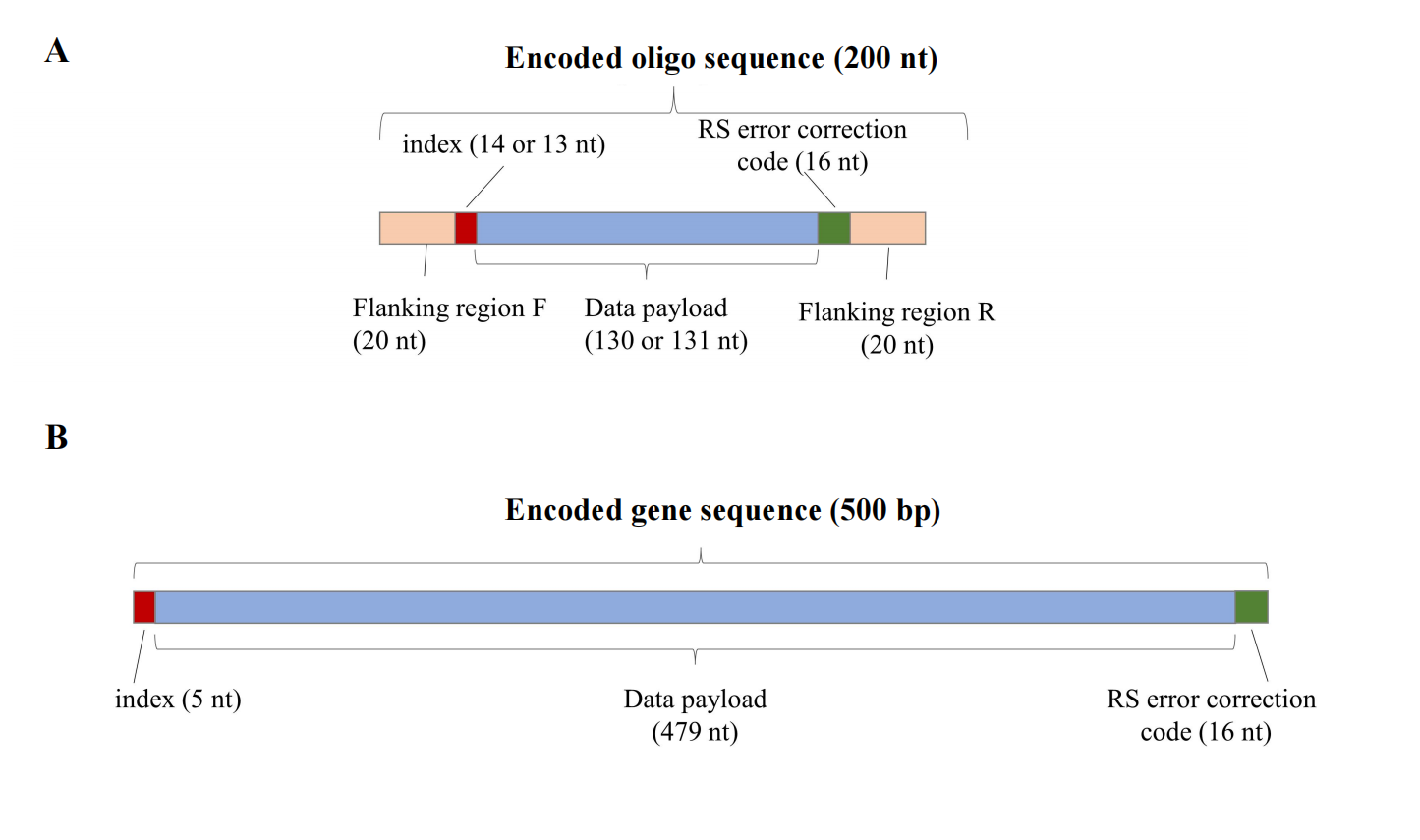


### Figure S6. The architecture of the encoded DNA sequence by “Wukong”.

(A) Encoded oligo sequenc; (B) Encoded gene sequence.


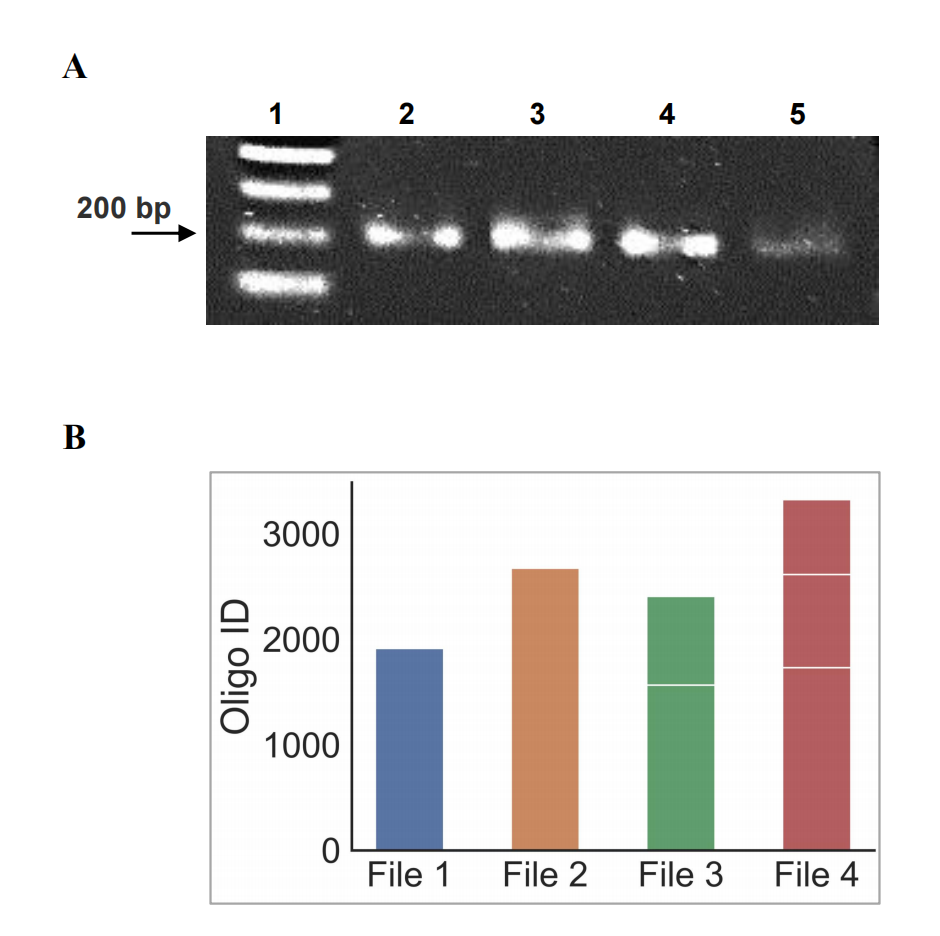


### Figure S7. Experimental validation of “Wukong” implemented in Storage-D.

(**A**) PCR amplification of each file from the synthesized DNA pool detected by agarose gel. Lane1, marker; Lane 2, Diagnosis and treatment protocol for COVID‐19 patients Trial Version 8; Lane 3, Diagnosis and treatment protocol for COVID‐19 patients Trial Version 9; Lane 4, Diagnosis and treatment protocol for COVID‐19 patients Trial Version 7; Lane 5, Treatise on Febrile and Miscellaneous Diseases. (**B**) The coverage of sequencing results towards the encoded sequence. The white line represents this encoded sequence was missing from the sequencing pool. The number on the horizontal axis represents sequence ID. File 1, 2 and 3 represents *Diagnosis and treatment protocol for COVID‐19 patients Trial Version* 7, 8 and 9; File 4, *Treatise on Febrile and Miscellaneous Diseases.*


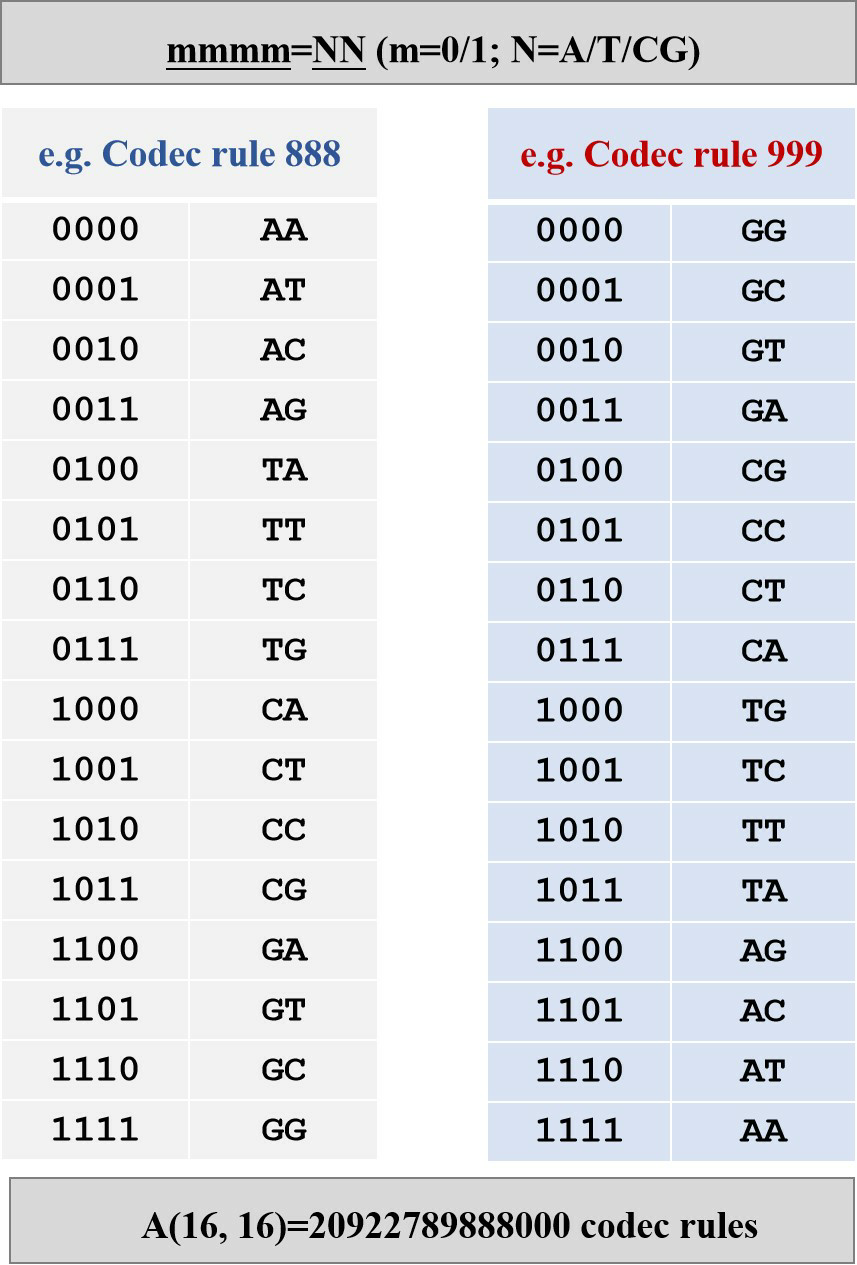


### **Figure S8. The mapping relationship between 0/1 bits and A/T/C/G sequence.**

Each binary bit unit with four bits are mapped to the DNA unit with two nucleotides. This generates 20, 922, 789, 888, 000 codec rules by a permutation combination. “Codec rule 888” and “Codec rule 999” represents two of these rules.


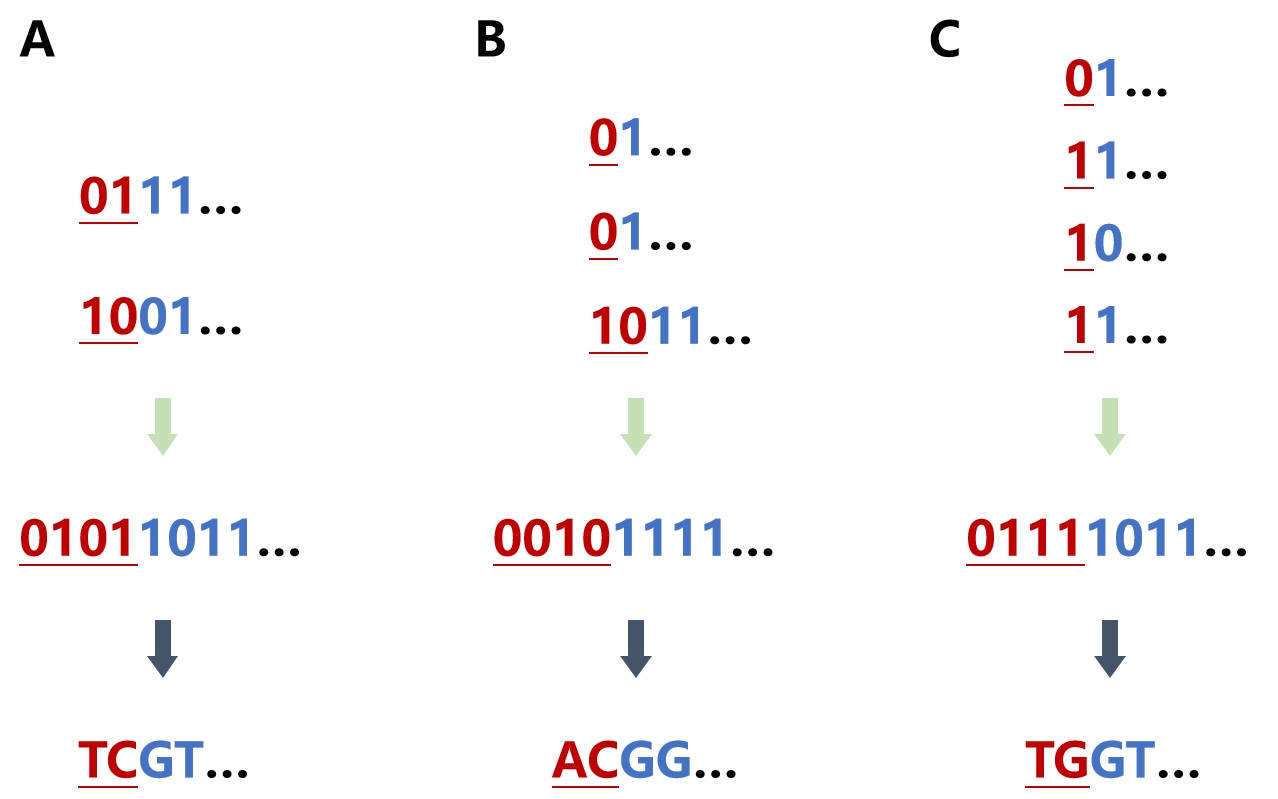


### Figure S9. Illustration of encoding different binary strings into one DNA sequence by “Wukong”.

(**A**) Two binary strings are encoded into one DNA sequence; (**B**) Three binary strings are encoded into one DNA sequence; (**C**) Four binary strings are encoded into one DNA sequence.

**01001000011001010110**

**11000110110001101111**

$$\bigoplus$$

**1000 1110 1010 0011 1001**

### Figure S10. Schematic overview of redundancy generation.

Two binary fragments are used to generate another binary fragment by an “XOR” conversion. XOR is frequently regarded as non carry addition as these laws are the same as addition but do not involve carry. The XOR operation is 0 ⊕ 0=0, 1 ⊕ 0=1, 0 ⊕ 1=1, 1 ⊕ 1=0 (both 0 and 1).


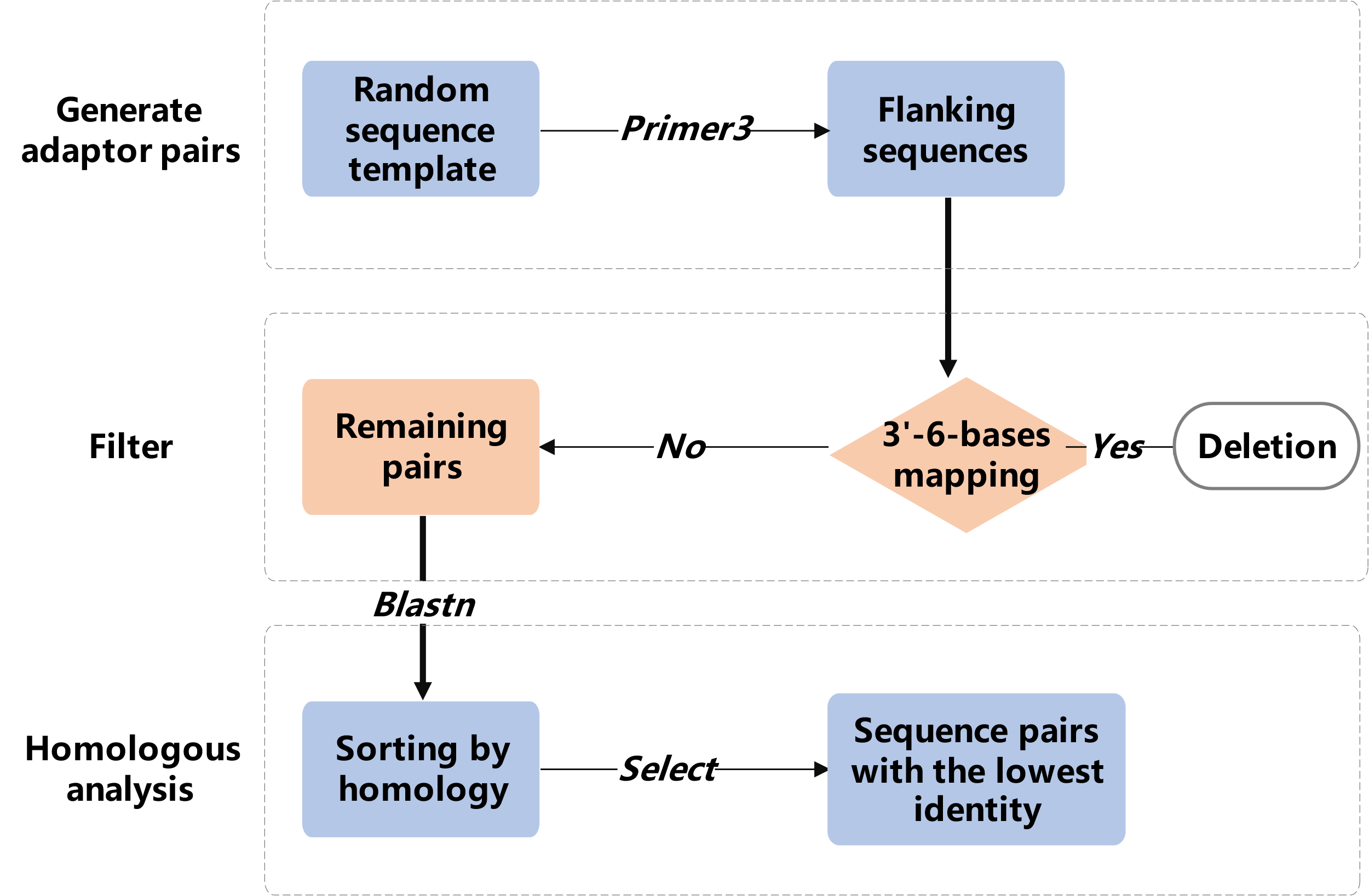


### Figure S11. The pipeline of random-access flanking sequence design.

Flanking sequences are firstly generated by the well-accepted primer-designing algorithm “Primer 3” [8, 9] by using random sequence as a template. Sequences with 6 nucleotides at 3’ end that are the same as the encoded DNA sequences are discarded. The rest of adaptor pair sequence undergoes a homologous analysis and the flanking sequence pairs with the lowest identity towards the encoded DNA sequence pool are selected.


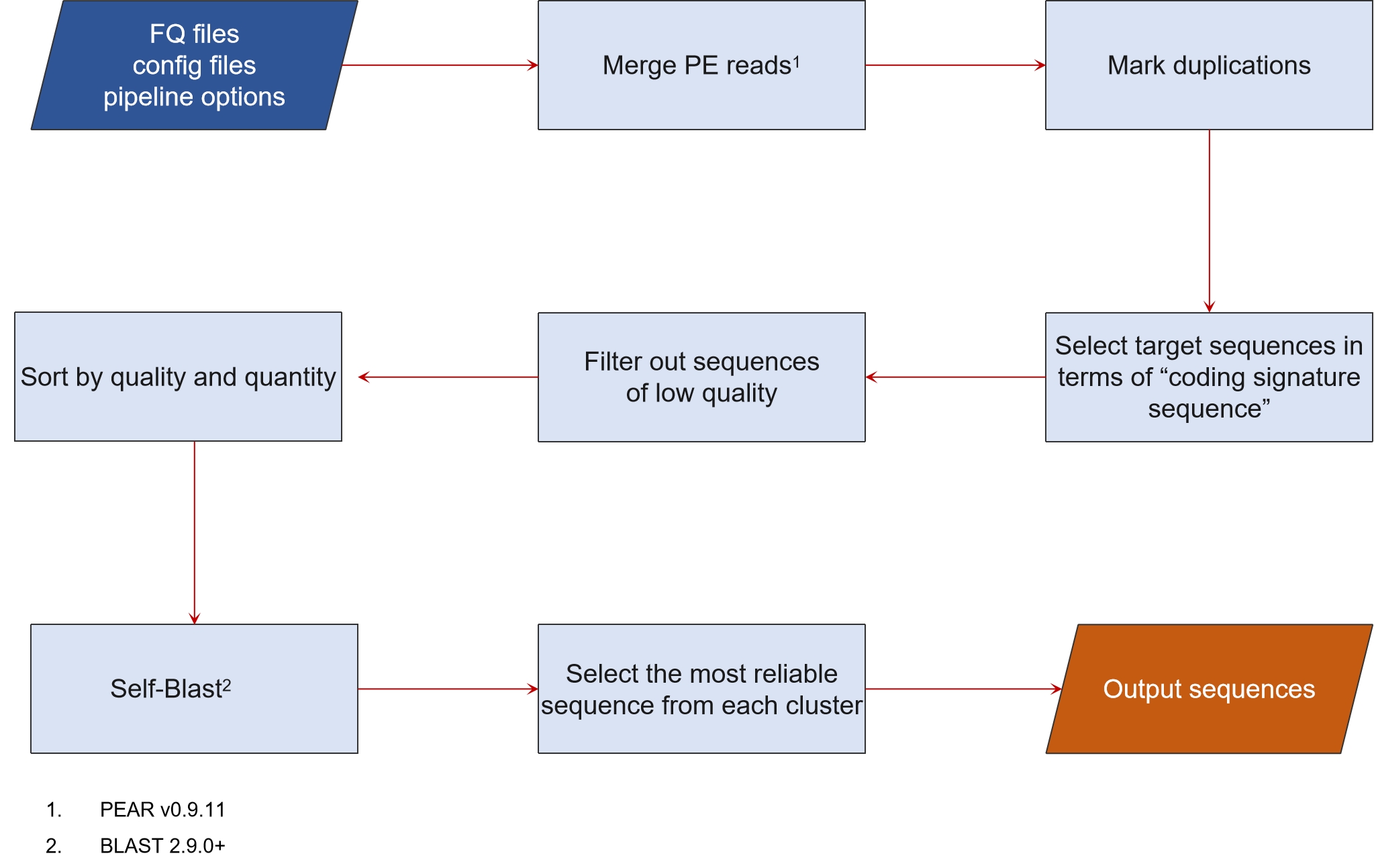


### Figure S12. The pipeline of sequencing data analysis.

This pipeline is suitable for pair-end sequencing of oligonucleotide library synthesized from encoded DNA sequence by different algorithms. The output sequences can be directly used for decoding analysis. The published algorithm, PEAR [10] is used for PE reads merging and the BLAST algorithm [11] from is employed for Self-BLAST analysis.

## References

1. Ping, Zhi, Shihong Chen, Guangyu Zhou, Xiaoluo Huang, Sha Joe Zhu, Haoling Zhang, Henry H. Lee, et al. 2022. “Towards practical and robust DNA-based data archiving using the yin–yang codec system.” *Nature Computational Science* 2: 234-242. https://doi.org/10.1038/s43588-022-00231-2

2. Grass, Robert N., Reinhard Heckel, Michela Puddu, Daniela Paunescu, Wendelin J. Stark. 2015. “Robust Chemical Preservation of Digital Information on DNA in Silica with Error-Correcting Codes.” *Angewandte Chemie-International Edition* 54: 2552-2555. <https://doi.org/10.1002/anie.201411378>

3. Erlich, Yaniv, Dina Zielinski. 2017. “DNA Fountain enables a robust and efficient storage architecture.” *Science* 355: 950-953. <https://doi.org/10.1126/science.aaj2038>

4. Press, William H., John A. Hawkins, Stephen K. Jones Jr, Jeffrey M. Schaub, Ilya J. Finkelstein. 2020. “HEDGES error-correcting code for DNA storage corrects indels and allows sequence constraints.” *Proceedings of the National Academy of Sciences of the United States of America* 117: 18489-18496. <https://doi.org/10.1073/pnas.2004821117>

5. Jeong, Jaeho, Seong-Joon Park, Jae-Won Kim, Jong-Seon No, Ha Hyeon Jeon, Jeong Wook Lee, Albert No, Sunghwan Kim, Hosung Park. 2021. “Cooperative sequence clustering and decoding for DNA storage system with fountain codes.” *Bioinformatics* 37: 3136-3143. <https://doi.org/10.1093/bioinformatics/btab246>

6. Choi, Yeongjae, Taehoon Ryu, Amos C. Lee, Hansol Choi, Hansaem Lee, Jaejun Park, Suk-Heung Song, et al. 2019. “High information capacity DNA-based data storage with augmented encoding characters using degenerate bases.” *Scientific Reports* 9: <https://doi.org/10.1038/s41598-019-43105-w>

7. Bettayeb, Meriem, Sara Ghunaim, Nour Mohamed, Nasir. 2019. “Error Correction Codes in Wireless Sensor Networks: A Systematic Literature Review.” *2019 3rd International Conference on Communications, Signal Processing, and Their Applications (Iccspa)* 1-6. <https://doi.org/10.1109/iccspa.2019.8713725>

8. Untergasser, Andreas, Ioana Cutcutache, Triinu Koressaar, Jian Ye, Brant C. Faircloth, Maido Remm, Steven G. Rozen. 2012. “Primer3-new capabilities and interfaces.” *Nucleic Acids Research* 40: e115-e115. <https://doi.org/10.1093/nar/gks596>

9. Untergasser, Andreas, Harm Nijveen, Xiangyu Rao, Ton Bisseling, René Geurts, Jack A.M. Leunissen. 2007. “Primer3Plus, an enhanced web interface to Primer3.” *Nucleic Acids Research* 35: W71-W74. <https://doi.org/10.1093/nar/gkm306>

10. Zhang, Jiajie, Kassian Kobert, Tomáš Flouri, Alexandros Stamatakis. 2014. “PEAR: a fast and accurate Illumina Paired-End reAd mergeR.” *Bioinformatics* 30: 614-620. <https://doi.org/10.1093/bioinformatics/btt593>

11. Ye, Jian, Scott McGinnis, Thomas L. Madden. 2006. “BLAST: improvements for better sequence analysis.” *Nucleic Acids Research* 34: W6-W9. <https://doi.org/10.1093/nar/gkl164>
